# Dissecting the role of *TP53* alterations in del(11q) chronic lymphocytic leukemia

**DOI:** 10.1002/ctm2.304

**Published:** 2021-02-04

**Authors:** Miguel Quijada‐Álamo, Claudia Pérez‐Carretero, María Hernández‐Sánchez, Ana‐Eugenia Rodríguez‐Vicente, Ana‐Belén Herrero, Jesús‐María Hernández‐Sánchez, Marta Martín‐Izquierdo, Sandra Santos‐Mínguez, Mónica del Rey, Teresa González, Araceli Rubio‐Martínez, Alfonso García de Coca, Julio Dávila‐Valls, José‐Ángel Hernández‐Rivas, Helen Parker, Jonathan C. Strefford, Rocío Benito, José‐Luis Ordóñez, Jesús‐María Hernández‐Rivas

**Affiliations:** ^1^ Cancer Research Center University of Salamanca, IBSAL, IBMCC, CSIC Salamanca Spain; ^2^ Department of Hematology University Hospital of Salamanca Salamanca Spain; ^3^ Department of Medical Oncology Dana‐Farber Cancer Institute Boston Massachusetts USA; ^4^ Broad Institute of Harvard and MIT Cambridge Massachusetts USA; ^5^ Department of Hematology Hospital Miguel Servet Zaragoza Spain; ^6^ Department of Hematology Hospital Clínico de Valladolid Valladolid Spain; ^7^ Department of Hematology Hospital Nuestra Señora de Sonsoles Ávila Spain; ^8^ Department of Hematology Hospital Universitario Infanta Leonor, Universidad Complutense Madrid Spain; ^9^ School of Cancer Sciences Faculty of Medicine University of Southampton Southampton UK; ^10^ Department of Medicine University of Salamanca Salamanca Spain

**Keywords:** biomarkers, chromosomal abnormality, chronic lymphocytic leukemia, CRISPR/Cas9 system, next‐generation sequencing, TP53 gene

## Abstract

**Background:**

Several genetic alterations have been identified as driver events in chronic lymphocytic leukemia (CLL) pathogenesis and oncogenic evolution. Concurrent driver alterations usually coexist within the same tumoral clone, but how the cooperation of multiple genomic abnormalities contributes to disease progression remains poorly understood. Specifically, the biological and clinical consequences of concurrent high‐risk alterations such as del(11q)/*ATM*‐mutations and del(17p)/*TP53*‐mutations have not been established.

**Methods:**

We integrated next‐generation sequencing (NGS) and clustered regularly interspaced short palindromic repeats (CRISPR)/Cas9 techniques to characterize the in vitro and in vivo effects of concurrent monoallelic or biallelic *ATM* and/or *TP53* alterations in CLL prognosis, clonal evolution, and therapy response.

**Results:**

Targeted sequencing analysis of the co‐occurrence of high‐risk alterations in 271 CLLs revealed that biallelic inactivation of both *ATM* and *TP53* was mutually exclusive, whereas monoallelic del(11q) and *TP53* alterations significantly co‐occurred in a subset of CLL patients with a highly adverse clinical outcome. We determined the biological effects of combined del(11q), *ATM* and/or *TP53* mutations in CRISPR/Cas9‐edited CLL cell lines. Our results showed that the combination of monoallelic del(11q) and *TP53* mutations in CLL cells led to a clonal advantage in vitro and in in vivo clonal competition experiments, whereas CLL cells harboring biallelic *ATM* and *TP53* loss failed to compete in in vivo xenotransplants. Furthermore, we demonstrated that CLL cell lines harboring del(11q) and *TP53* mutations show only partial responses to B cell receptor signaling inhibitors, but may potentially benefit from ATR inhibition.

**Conclusions:**

Our work highlights that combined monoallelic del(11q) and *TP53* alterations coordinately contribute to clonal advantage and shorter overall survival in CLL.

## Graphical abstract

1. Combined del(11q) and *TP53* alterations are related to a highly adverse outcome in CLL, and their co‐occurrence leads to clonal advantage in vitro and in vivo.

2. Biallelic *ATM* and *TP53* alterations are mutually exclusive, showing impaired proliferative capacity in cellular models and a deficient engraftment in vivo.

3. Del(11q) *TP53* ^MUT^ cells show partial responses to BCR signaling inhibitors and may potentially benefit from ATR inhibition.

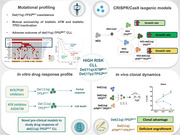

**Abbreviations**
ATR: ataxia telangiectasia and Rad3 related
BCR: B cell receptor
CLL: chronic lymphocytic leukemia
CRISPR: clustered regularly interspaced short palindromic repeats
DDR: DNA damage response
FACS: fluorescence‐activated cell sorting
FISH: fluorescence in situ hybridization; GFP, green fluorescent protein
iwCLL: international workshop on CLL
MACS: magnetically activated cell sorting
MTT: (3‐(4,5‐dimethylthiazol‐2‐yl)‐2,5‐diphenyltetrazolium bromide
NGS: next‐generation sequencing
OS: overall survival
PBS: phosphate‐buffered saline
WES: whole exome sequencing studies

## 1 BACKGROUND

Chronic lymphocytic leukemia (CLL) is a clonal B‐cell malignancy characterized by an extremely variable clinical course.^1^, ^2^ This variability is a result of the underlying biological heterogeneity, highlighted by the presence of genomic alterations that play an important role in disease prognosis.^3^, ^4^, ^5^ Monoallelic deletion of chromosome 11q22.3 (del(11q)) is present in 15–20% of CLL cases at diagnosis, and it is considered a high‐risk cytogenetic alteration in CLL.^3^, ^6^, ^7^ The size of this deletion is variable, with a commonly minimal deleted region almost always encompassing the *ATM* gene.^8^, ^9^, ^10^ *ATM* has a key role in cell‐cycle checkpoint activation and the DNA damage response (DDR) pathway.^11^ Although the presence of this deletion is associated with poor outcomes,^3^, ^12^ del(11q) CLL exhibits considerable clinical heterogeneity,^13^ suggesting that other concomitant genetic alterations may have a role in the prognosis of CLL patients from this high‐risk subgroup.^14^ Indeed, it has been reported that monoallelic *ATM* deletion is not enough to cause a CLL‐like disease in mice.^15^ Deleterious mutations in the remaining allele of *ATM* or gain‐of‐function *SF3B1* mutations in del(11q) patients have been shown to drive CLL oncogenesis, being associated with a worse prognosis.^15^, ^16^, ^17^, ^18^, ^19^ Nevertheless, these mutations only account for 30% or 20% of del(11q) cases, respectively, leaving more than half of del(11q) patients with unknown second driver of the disease.

Another high‐risk‐associated cytogenetic feature in CLL is the deletion of chromosome 17p13.1 (del(17p)), which appears in 4–10% of CLL cases at diagnosis^20^, ^21^ and in up to 40% of relapsing cases to chemoimmunotherapy.^22^, ^23^ Del(17p) invariably comprises the tumor suppressor gene *TP53*, which is mutated in ∼10% of CLL cases at diagnosis and plays a critical role in the DDR pathway, apoptosis and cell cycle.^24^ In addition, *TP53* gene mutations in the remaining allele of del(17p) patients have been found in 80% of these cases,^25^ indicating that biallelic dysfunction of this gene appears to be the main mechanism driving CLL progression and relapse in this subgroup CLL patients.^20^, ^26^

Some studies have reported that biallelic *ATM* abnormalities appear in low co‐occurrence with *TP53* lesions, suggesting that the complete dysfunction of any of these proteins cannot coexist with the defect of the other.^20^, ^27^ On the contrary, monoallelic del(11q) or *ATM* mutations have been associated in some cases with monoallelic *TP53* defects, suggesting that monoallelic abnormalities in both of these genes may be co‐selected during the disease evolution.^20^, ^28^ However, these observations have only been made through FISH or next‐generation sequencing (NGS) studies. There lacks further research investigating the biological effects of combined monoallelic or biallelic *ATM* and *TP53* alterations in CLL. Recent work by our group and others has implemented the clustered regularly interspaced short palindromic repeats (CRISPR)/Cas9 technology for the efficient generation of CLL driver gene mutations or chromosomal abnormalities in human cell lines and animal models,^15^, ^29^, ^30^, ^31^, ^32^ offering new opportunities to study the potential co‐operative effects of concurrent genetic alterations in CLL as well as the biological basis of mutual exclusivity among CLL driver alterations.

In this study, we used targeted sequencing and CRISPR/Cas9 approaches to systematically characterize the biological effects of concurrent monoallelic or biallelic *ATM* and/or *TP53* lesions in CLL cells in relation to patient clinical outcome. We showed that *TP53* alterations are a robust prognostic marker for overall survival in monoallelic del(11q) patients, whereas biallelic inactivation of both of these genes occurs in a mutually exclusive way. We functionally validated these findings with isogenic CRISPR/Cas9 generated models of del(11q) together with *TP53* and/or *ATM* mutations, showing that CLL cell lines harboring biallelic loss of *ATM* and *TP53* present abnormal cell cycle and mitotic profiles, failing to engraft and compete in murine xenotransplants. These results offer a functional insight into the mutual exclusivity of these alterations in CLL. Conversely, *TP53* mutations conferred a clonal advantage of CLL cells with a monoallelic del(11q) background, being able to outgrow cells with single del(11q) in in vivo clonal competition experiments.

## 2 METHODS

### 2.1 Patients

This study was based on 271 well‐characterized CLL cases, diagnosed according to the international workshop on CLL (iwCLL) guidelines, with clinical data and FISH information. Overall, this cohort was enriched in del(11q)/del(17p) cases in concordance to the main purpose of the study. Forty‐seven patients harbored del(11q), and the rest of them (*n* = 224), designated as the control group, were representative of the entire CLL cohort in terms of demographic and clinical‐biologic characteristics. At the time of analysis, 94% of patients were untreated and 50% of them received treatment during follow‐up. The median follow‐up was 60 months (range 0–264 months), and the median time to first treatment (TFT) was 18.5 months (range 0–221 months). Clinical and biological characteristics of the 271 CLL patients are summarized in Table 1.

**TABLE 1:** Clinical and biological characteristics of CLL patients

			**Non del(11q) (*n *= 224), %**	**del(11q) (*n *= 47), %**	**p value**
Male			63.8	73.7	0.239
Age of diagnosis (median, range)			73 (38‐97)	69 (43‐97)	0.152
Binet stage B/C			24.0	41.3	**0.016**
*IGHV* unmutated			38.1	81.0	**<0.001**
CD38 positivity			25.3	44.8	**0.034**
Hepatomegaly			3.9	11.4	**0.046**
Splenomegaly			15.6	27.3	0.066
FISH cytogenetics					
	Del(13q)		62.1	48.9	0.095
	Trisomy 12		21.4	2.2	**0.002**
	Del(17p)		7.6	25.5	**0.0003**
Next‐generation sequencing					
	Mutated gene		75.4	92.5	**0.014**
	*ATM* ^MUT^		4.9	34.0	**<0.001**
	*NOTCH1* ^MUT^		20.5	17.0	0.574
	*SF3B1* ^MUT^		10.3	17.0	0.19
	*BIRC3* ^MUT^		3.6	19.1	**< 0.001**
	*TP53* ^MUT^		12.1	23.4	**0.043**
Monoallelic *TP53* alteration (% del(17p)/ %*TP53* ^MUT^)			10.3 (2.7/7.6)	14.9 (8.5/6.4)	0.413
Biallelic *TP53* alteration (del(17p) and *TP53* ^MUT^)			4.5	17.0	**0.002**

Samples and clinical data were collected from 16 Spanish institutions. This study was approved by the local ethical committee (Comité Ético de Investigación Clínica, Hospital Universitario de Salamanca). Written informed consent was obtained from all participants before they entered the study. In addition, 136 CLL patients (*n* del(11q) = 38) from an independent cohort were included for external validation.^10^

### 2.2 NGS

Mutational analysis was performed in the whole cohort of CLL patients included in this study (*n *= 271). Genomic DNA was isolated from peripheral blood or bone marrow MACS‐sorted CD19+ B‐lymphocytes. The Agilent SureSelectQXT Target Enrichment system for Illumina Multiplexed Sequencing (Agilent Technologies, Santa Clara, CA, USA) was used to produce libraries of exonic regions from 54 CLL‐related genes included in a custom‐designed panel previously validated^33^ (Table S1). Genomic DNA extracted from each sample was fragmented and used to construct a sequencing library with the SureSelectQXT Library Prep Kit following the manufacturer's instructions. Paired‐end sequencing (151‐bp reads) was run on the Illumina NextSeq instrument (Illumina, San Diego, CA, USA). Median coverage of target regions was 600 reads/base, with at least 100X in 97% of them. Data analysis and variant calling were performed using the bioinformatic pipelines described in the Supplementary Methods.

### 2.3 CRISPR/Cas9‐mediated mutagenesis in CLL cell lines

The constitutive Cas9 expression vector (LentiCas9‐Blast, Addgene #52962) was used to generate HG3 cell lines stably expressing the protein Cas9. Lentiviral particles containing plasmid‐expressing Cas9 and blasticidine resistance were transduced into each cell line and selected by blasticidine (20 μg/ml) for 2 weeks.^34^ Cas9 activity was tested using a previously reported system based on the pXPR‐011 plasmid (Addgene #59702) which delivers green fluorescent protein (GFP) and RNA guide (sgRNA) targeting GFP.^35^

SgRNAs were designed using the online CRISPR design tool (http://crispr.mit.edu/) to target *TP53* (exon 4) and/or *ATM* (exon 10). These exons were chosen for editing in order to generate frameshift mutations that result in a complete deletion of the DNA Binding Domain of TP53 or the FAT and PIKK C‐terminal domains of ATM,^11^, ^25^ producing a protein disruption as a consequence. The selection of the sgRNAs was based on choosing those of highest efficiency to target the gene of interest and with the lowest predicted off‐targets effects. In addition, a sgRNA designed not to target the human genome was used as a negative control. Sequences of the selected sgRNAs are detailed in Table S2. The procedure and sgRNAs used for the generation of del(11q) were previously described.^32^ SgRNAs targeting *TP53* were cloned into pLKO5.sgRNA.EFS.GFP (Addgene_#57822) or pLKO5.sgRNA.EFS.tRFP (Addgene_#57823), and sgRNAs targeting *ATM* into pLKO5.sgRNA.EFS.tRFP (Addgene_#57823). Negative control sgRNA was cloned in both vectors. Cloning, transduction, and clone screening are detailed in Supplementary Methods.

The presence of CRISPR/Cas9‐mediated off‐target indels was examined in each generated clone by analyzing the top #3 gene off‐targets of each sgRNA designed using Sanger sequencing. The Off‐Spotter online tool was used to predict the off‐target regions for each sgRNA (https://cm.jefferson.edu/Off-Spotter/).^36^ Off‐target regions, number of mismatches and primers designed for sequencing are detailed in Table S3.

### 2.4 In vivo clonal competition and survival experiments

Animal studies were conducted in accordance with the Spanish and European Union guidelines for animal experimentation (RD53/2013, Directive‐2010/63/UE, respectively) and received prior approval from the Bioethics Committee of our institution.

For the in vivo clonal competition experiments, a total of 16 4‐ to 5‐week‐old female NSG mice were previously randomized and used for intravenous injection of pooled HG3^WT^ + HG3 *TP53* ^MUT^ cells (1.25 × 10^6^ cells/mice; ratio 1:1; *n* = 8) and HG3‐del(11q) + HG3‐del(11q) *TP53* ^MUT^ + HG3‐del(11q) *ATM* ^MUT^ *TP53* ^MUT^ (1.25 × 10^6^ cells/mice; ratio 1:1:1; *n* = 8). Mice were sacrificed 7 and 14 days after cell injection (*n* = 4/condition each time point), and spleens and bone marrow were collected for engraftment and clonal evolution analysis. Red blood cells were lysed with erythrocyte lysis buffer, and the remaining cells were then washed twice in phosphate‐buffered saline (PBS). Samples were subjected to FACS analysis on a FACSAria flow cytometer. Data were analyzed with FlowJo software based on GFP and/or RFP positivity: HG3^WT^ (GFP^+^RFP^−^), HG3 *TP53* ^MUT^ (GFP^−^RFP^+^); HG3‐del(11q) (GFP^−^RFP^+^), HG3‐del(11q) *TP53* ^MUT^ (GFP^+^RFP^−^), HG3‐del(11q) *ATM* ^MUT^ *TP53* ^MUT^ (GFP^+^RFP^+^).

For the survival analysis, 1.25 × 10^6^ cells of HG3^WT^, HG3 *TP53* ^MUT^, HG3‐del(11q), HG3‐del(11q) *TP53* ^MUT^, and HG3‐del(11q) *ATM* ^MUT^ *TP53* ^MUT^ clones were injected individually into NSG mice (n = 5/group). Peripheral blood was extracted at day 17 post‐injection for the assessment of engraftment by flow cytometry. Mice were monitored daily during the experiment and euthanized by anesthesia overdose when they presented symptoms of severe disease.

### 2.5 Statistics

Statistical analyses were performed using IBM SPSS for Windows, Version 23.0 (IBM Corp.) or GraphPad Prism software v6 (GraphPad Software). Data are summarized as the mean ± standard deviation (SD). Student's *t*‐test, Mann‐Whitney, ANOVA, and Kruskal‐Wallis tests were used to determine statistical significance. The chi‐square test was used to assess associations between categorical variables. Variables significantly associated with OS were identified by the Kaplan‐Meier method, and the curves of each group were compared with the log‐rank test. p values lower than 0.05 were considered as statistically significant.

## 3 RESULTS

### 3.1 Monoallelic del(11q) can co‐occur with *TP53* alterations while biallelic *ATM* and *TP53* alterations are mutually exclusive in CLL patients

**FIGURE 1:**
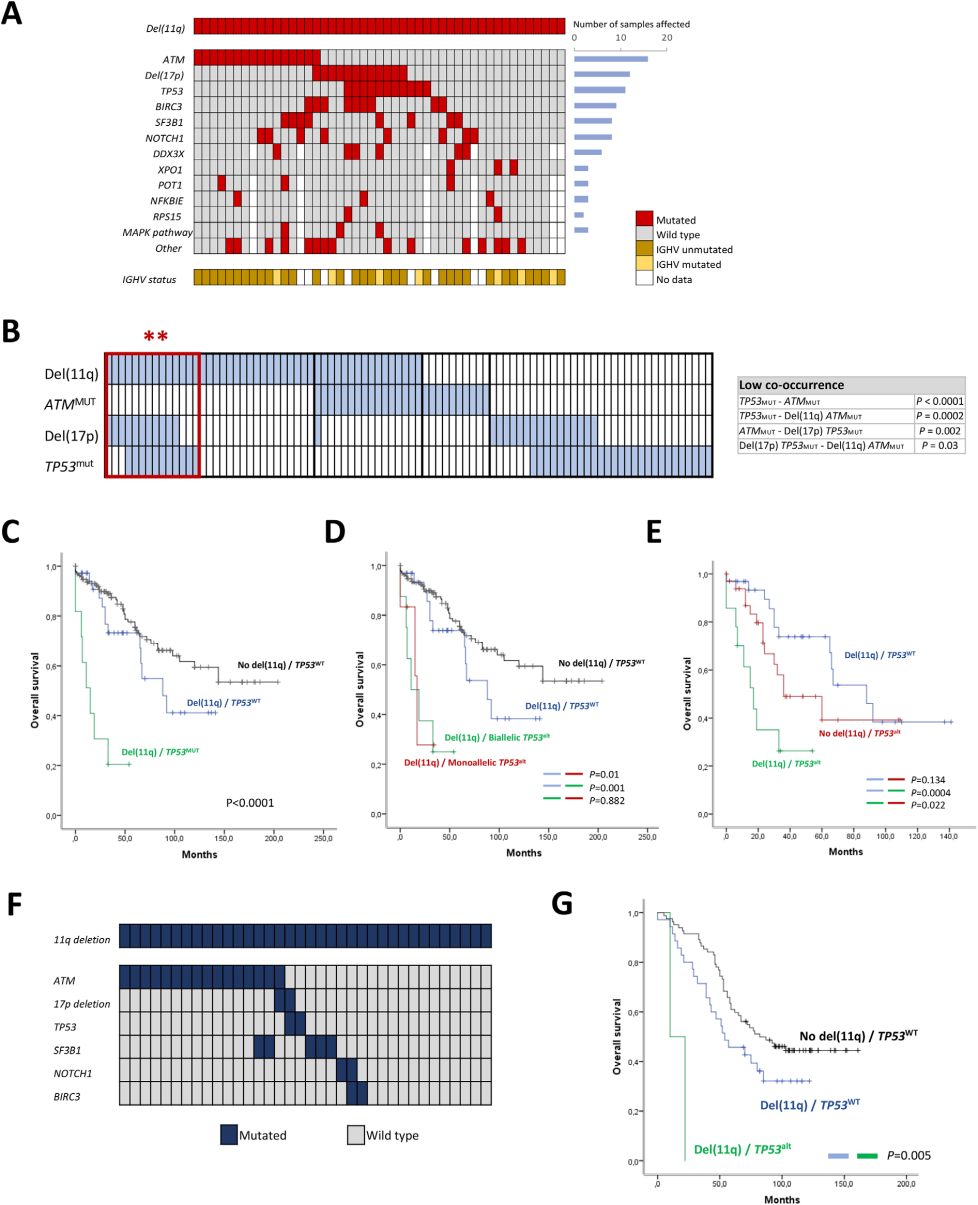
Mutational analysis and overall survival (OS) of del(11q) patients. (A) Mutational landscape of del(11q) patients; each column represents a patient and each row corresponds to a genetic alteration. Mutation or cytogenetic events are indicated in red, IGHV unmutated status in dark yellow, and IGHV mutated status in light yellow. White indicates missing data. (B) Significantly high and low co‐occurrences of *ATM* and *TP53* alterations in CLL patients (chi‐square test). Each column corresponds to one patient, and the presence of mutations or deletions is clustered according to the type of *ATM* and *TP53* alterations, shown in blue. Left red rectangle indicates the presence of high co‐occurrence between monoallelic del(11q) and *TP53* alterations (** p < 0.01). Right table indicates the grades of low co‐occurrences between the indicated conditions. (C) Impact of *TP53* mutations in the survival of del(11q) CLL patients. (D) OS of del(11q) patients according to the presence of monoallelic or biallelic *TP53* alterations (deletion and/or mutation). (E) Clinical impact of the presence of del(11q) in patients with *TP53* alterations (deletion and/or mutations). (F) Mutational analysis and (G) overall survival (OS) of del(11q) patients in the validation cohort from the UK LRF CLL4 trial

A total of 271 patients were included in this study: 47 del(11q) cases and 224 non‐del(11q). Mutational analyses showed that 93.6% (44/47) of del(11q) patients harbored a mutation in at least one of the 54 genes analyzed, whereas 75.4% (169/224) were mutated within the control group (p = 0.014) (Table 1). High mutational frequencies in *ATM* (34%), *TP53* (23.4%), *BIRC3* (19%), *SF3B1* (17%), and *NOTCH1* (17%) were observed in del(11q) patients (Figure 1A), being *ATM*, *BIRC3*, and *TP53* mutations significantly associated with del(11q) (p < 0.0001, p < 0.0001, p = 0.043, respectively) (Table 1).

*TP53* alterations (deletions and/or mutations) were one of the most abundant genetic lesions in our del(11q) series (15/47, 31.9%), after *ATM* mutations (16/47, 34%) (Figure 1A). Within this subgroup, a total of 12 patients had del(17p), and eight of them exhibited a *TP53* mutation in the remaining allele (67%), therefore considered to have a biallelic *TP53* alteration. In addition, *TP53* mutations were detected in three del(11q) patients without del(17p). Regarding the type of the genetic mutations in patients with del(11q) and del(17p), we found that four of them were frameshift, one encoded for a stop codon, and nine were missense mutations, while mutations in del(11q) patients without del(17p) were all missense substitutions (Table 2).

**TABLE 2:** TP53 alterations identified in del(11q) CLL patients

**Patient ID**	**del(11q), % of cells**	***TP53* alterations (del/mut)**	**del(17p), % of cells**	***TP53* mutations, AA change (Transcript: NM_000546.5)**	**VAF, %**	**Other mutated genes**
**16**	92	del/wt	86	–	–	*ATM, BIRC3, ZMYM3, SETD2*
**17**	50	del/wt	34	–	–	*BIRC3, NOTCH1, CHD2, BCOR*
**18**	20.5	del/wt	18	–	–	*CHD2*
**19**	90	del/wt	22	–	–	*KRAS*
**20**	15	del/mut	59	R273L; I332fs; M384fs	4.75; 3.09; 2.86	*BIRC3, RPS15, DDX3X*
**21**	62	del/mut	24	L330R	6.26	*BIRC3, DDX3X*
**22**	89	del/mut	87	P278R	98.96	*BIRC3, NFKBIE*
**23**	48.5	del/mut	38	R342X	15.33	*BIRC3*
**24**	62	del/mut	65	R273L; G105fs; P152L	33.96; 7.91; 3.77	*SF3B1, MAP2K1, BRAF, DDX3X, FUBP1, ZC3H18*
**25**	50	del/mut	25	R290H; Y163C	42.35; 11.92	*NOTCH1, ZMYM3, ASXL1, FAT1*
**26**	54	del/mut	51	I195T	20.72	*–*
**27**	46	del/mut	38	L130V; L93fs	21.9; 13.53	*MGA*
**28**	43	wt/mut	–	L145P	65.57	*SF3B1, ARID1A, ASXL1, MED12*
**29**	10	wt/mut	–	Y234C	3.33	*–*
**30**	83	wt/mut	–	Y205D	7	*–*

Abbreviations: fs, frameshift mutation; VAF, variant allele frequency.

In addition to the mutational analysis, we also focused on the co‐occurrence of del(11q)/*ATM* mutations and del(17p)/*TP53* mutations in CLL patients, according to the presence of monoallelic or biallelic alteration and the type of the genetic alteration: deletion or gene mutation. First, regarding only mutations, we detected a significantly low co‐occurrence between *ATM* and *TP53* mutations (p < 0.0001). Second, considering also chromosomal deletions in the analysis, we found a significant lack of *ATM* mutations in patients with biallelic *TP53* alterations (deletion and mutation) (p = 0.002) as well as a complete absence of *TP53* mutations in patients with biallelic *ATM* inactivation (p = 0.0002) (Figure 1B). We also detected a mutual exclusivity between biallelic *TP53* and biallelic *ATM* alteration (p = 0.03). Conversely, we observed a statistically significant association between *TP53* alterations (del(17p) and/or *TP53* mutation) and monoallelic *ATM* loss by del(11q) (p = 0.0002) (Figure 1B). Altogether, these results indicate that del(11q) CLL cells may harbor additional *TP53* alterations as long as they have the remaining *ATM* ^WT^ allele intact.

### 3.2 CLL patients harboring combined del(11q) and *TP53* alterations exhibit a highly adverse outcome

We next analyzed whether mutations in the most frequently mutated genes in del(11q) patients had an impact on their survival. Notably, only *TP53* mutations were able to stratify del(11q) patients in terms of clinical impact: those patients with *TP53* mutations had shorter OS than the rest of del(11q) (median 15 vs 88 months, p < 0.0001) (Figure 1C; Table S4). In addition, no significant differences regarding OS were observed between del(11q) patients harboring mutations in the rest of the genes analyzed by targeted NGS (Table S4; Figure S1).

Given the clinical impact of *TP53* mutations in the OS of del(11q) patients, we performed a clinical analysis with respect to the number of alleles affected by *TP53* alterations. Regarding the entire CLL cohort, we observed that patients harboring a biallelic *TP53* inactivation showed a significantly shorter overall survival than those with monoallelic *TP53* alteration (median 19 vs 60 months, p = 0.016) (Figure S2A).

Within del(11q) cases, the median OS of patients with biallelic *TP53* alteration was significantly shorter than del(11q) *TP53* ^WT^ patients (median 11 vs 88 months, p = 0.001) (Figure 1D). Besides, we detected differences between the median OS of del(11q) cases harboring monoallelic *TP53* alterations by either del(17p) or somatic mutation and the rest of del(11q) patients (median 17 vs 88 months, p = 0.01) (Figure 1D). Interestingly, no differences in OS were observed between biallelic and monoallelic del(11q) *TP53* ^ALT^ patients (median 11 vs 17 months, p = 0.882), suggesting that the highly negative clinical impact of this co‐occurrence is independent of the number of alleles affected by *TP53* aberrations. In addition, patients with combined del(11q) and *TP53* abnormalities had a shorter TFT than those with only *TP53* alterations (median 7 vs 36 months, p = 0.05), while there were no significant differences with respect to the presence of sole del(11q) abnormality (median 7 vs 11 months, p = 0.33) (Figure S2B).

Furthermore, the presence of del(11q) also contributed to a shorter OS for patients with *TP53* alterations in our study (median 17 vs 36 months, p = 0.022), corroborating that the coexistence of both del(11q) and *TP53* alterations accounts for a marked poor outcome even in del(17p) cases (Figure 1E). These results were validated in cases from a previously published cohort from the UK LRF CLL4 trial (Figure 1F),^10^ where *TP53* alterations also accounted for a reduced survival of del(11q) patients (median 10 vs 54 months, p = 0.005; Figure 1G).

### 3.3 Concurrence of biallelic *ATM* and *TP53* alterations in CLL cells results in defective mitosis and the formation of abnormal multinucleated cells

**FIGURE 2:**
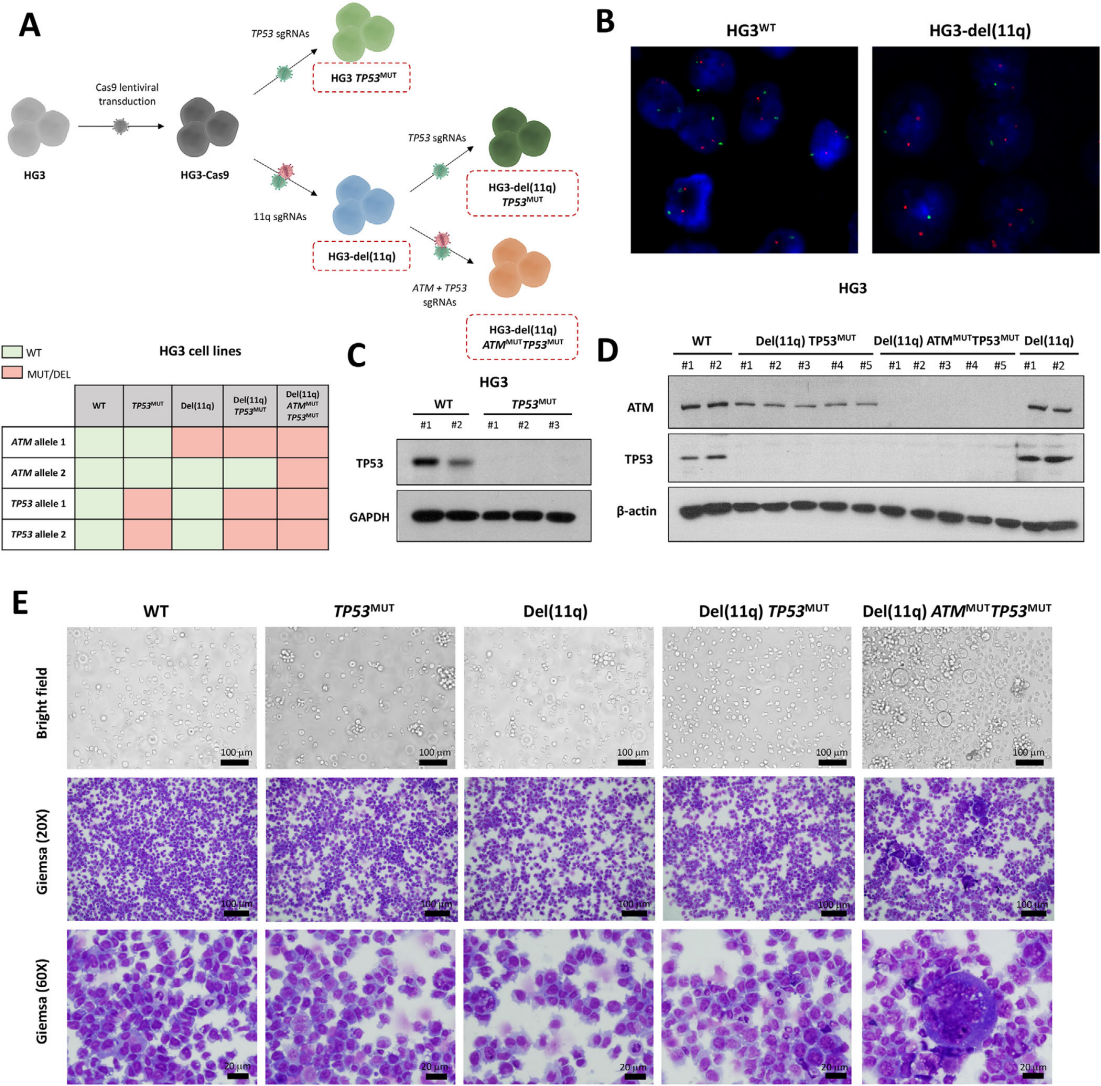
Generation of CRISPR/Cas9‐edited CLL cell lines harboring del(11q), TP53 and/or ATM mutations and phenotypical analysis of edited cells. (A) Upper panel: experimental design for the introduction of del(11q), *TP53*, and *ATM* mutations in the HG3 CLL derived cell line using the CRISPR/Cas9 system. sgRNAs targeting 11q22.1 and 11q23.3 were nucleofected for transitory expression in HG3‐Cas9 cells. Nucleofected single‐cell sorted clones were screened for the presence of del(11q), and the presence of this deletion was validated by Sanger sequencing and FISH. The resulting HG3‐del(11q) isogenic cell line, as well as parental HG3‐Cas9 cells, was further transduced with sgRNAs targeting *TP53* and/or *ATM* genes for the generation of truncating mutations. In total, 3 HG3^WT^, 3 HG3 *TP53* ^MUT^, 3 HG3‐del(11q), 5 HG3‐del(11q) *TP53* ^MUT^, and 5 HG3‐del(11q) *ATM* ^MUT^ *TP53* ^MUT^ clones were generated. Lower panel: number of alleles affected by mutations and deletions in the CRISPR/Cas9‐generated cell lines. (B) Representative FISH images of HG3^WT^ and HG3‐del(11q) cells. Green signals correspond to 11q22/ATM probe and the control red signals correspond to 17p13/TP53 probe. (C) Western blot analysis of isogenic HG3‐edited clones with *TP53* mutations. Upper panel shows TP53 protein expression of 2 HG3^WT^ clones and 3 HG3 *TP53* ^MUT^ clones. Lower panel shows GAPDH, which was used as loading control. (D) Western blot analysis of HG3‐edited single‐cell clones. Upper and middle panels show ATM and TP53 expression, respectively, of 2 HG3^WT^ clones, 5 HG3‐del(11q) *TP53* ^MUT^ clones, 5 HG3‐del(11q) *TP53* ^MUT^ *ATM* ^MUT^ clones, and 2 HG3‐del(11q) clones. β‐actin was used as loading control. (E) Bright field and Giemsa stained representative images of HG3‐edited cell lines

Based on the sequencing results observed in our CLL cohort, we next prompted to address the biological implications of concurrent monoallelic or biallelic loss of *ATM* and *TP53* in del(11q) CLL cells. For this purpose, we used the HG3 CLL derived cell line, which is diploid for chromosomes 11 and 17 and also has wild‐type *ATM* and *TP53* genes. HG3 parental cells were transduced to stably express Cas9 protein, and sgRNAs targeting chromosome 11q22.1/11q23.3 were introduced to generate an isogenic HG3 CLL cell line harboring del(11q) (HG3‐del(11q)) of ∼17 Mb size including *ATM* gene (Figures 2A and 2B).^32^ We then introduced sgRNAs targeting *ATM* and/or *TP53* genes in both wild‐type HG3 cells (HG3^WT^) and HG3‐del(11q) cells. Single‐cell FACS sorted clones were expanded and screened for the presence of truncating mutations in *TP53* and *ATM*. In total, we generated 3‐5 different single‐cell clones of the following conditions: HG3^WT^, HG3 *TP53* ^MUT^ (biallelic *TP53* truncating mutation), HG3‐del(11q) (monoallelic *ATM* loss), HG3‐del(11q) *TP53* ^MUT^ (monoallelic *ATM* loss / biallelic *TP53* truncating mutation), and HG3‐del(11q) *ATM* ^MUT^ *TP53* ^MUT^ (biallelic *ATM* loss / biallelic *TP53* truncating mutation) (Figure 2A). The type of CRISPR/Cas9‐mediated indels of *TP53* and/or *ATM* in each generated clone is specified in Table S5, and the functional absence of these proteins was validated by western blot (Figures 2C and 2D). In addition, no off‐target indels were found in any of the HG3‐edited clones (Table S5).

**FIGURE 3:**
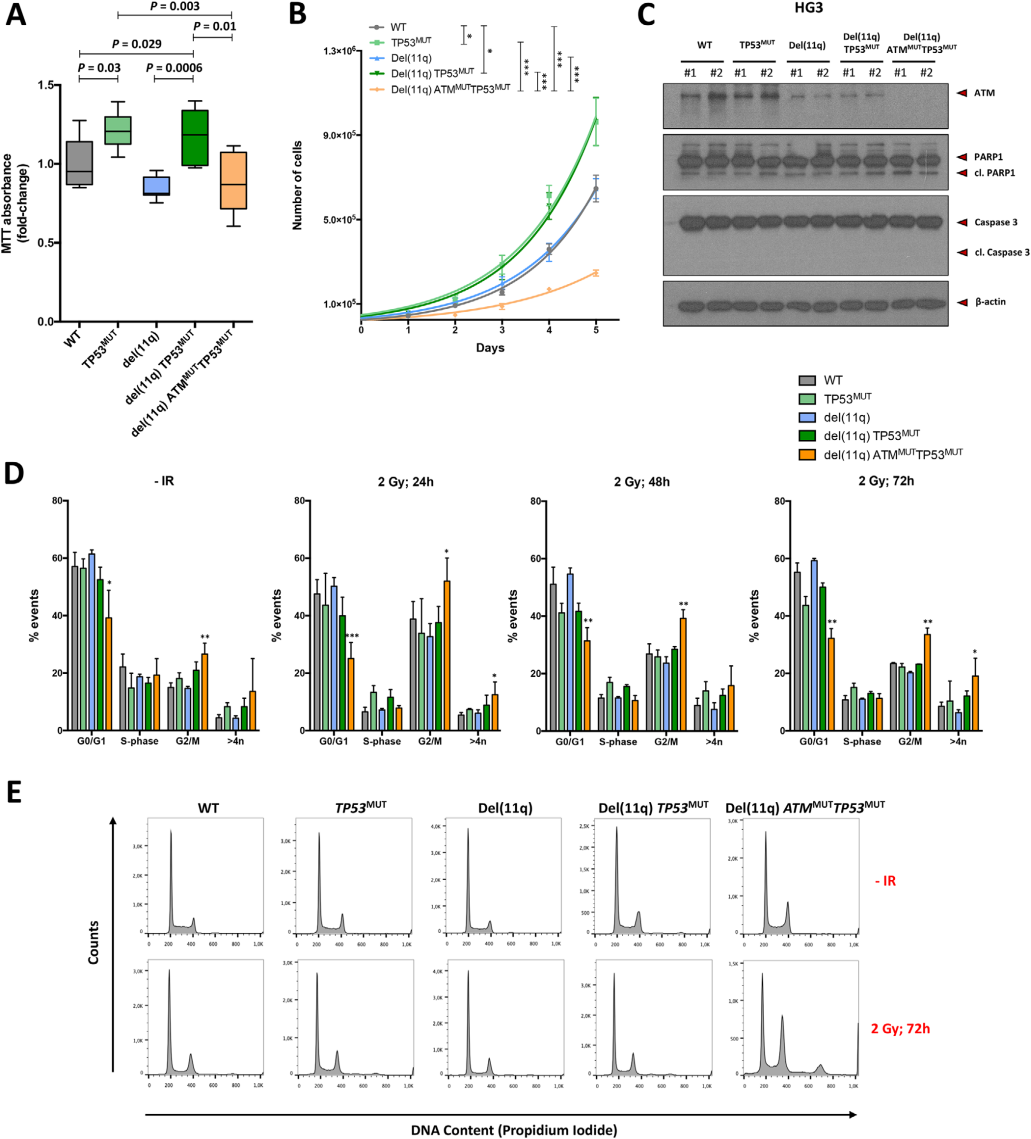
Effects of biallelic loss of *TP53* and *ATM* on viability, cell growth, apoptosis, and cell cycle control of CRISPR/Cas9‐edited cell lines. (A) Effects of del(11q), *TP53*, and/or and *ATM* mutations on proliferation of HG3 cells after 72 hours. MTT absorbance values are normalized with the HG3^WT^ clones. Data are summarized as the mean ± SD. (B) HG3‐edited cell lines were seeded at a concentration of 3 × 10^4^ cells/mL, and cell growth was assessed at five time points every 24 hours by Trypan Blue exclusion. Data were fitted in an exponential growth equation, and time point values are presented as the mean ± SEM. (C) Representative immunoblot analyses of HG3^WT^, HG3 *TP53* ^MUT^, HG3‐del(11q), HG3‐del(11q) *TP53* ^MUT^, and HG3‐del(11q) *ATM* ^MUT^ *TP53* ^MUT^ whole cell lysates. ATM, PARP1, and Caspase‐3 protein expression and/or cleavage were analyzed. β‐actin was used as loading control. (D) Cell cycle phase distribution of HG3‐edited cell lines upon exposure to irradiation at the indicated time points. Data represent the mean values ± SD of at least three independent experiments. p < 0.05 (*), p < 0.01 (**). (E) Representative cell cycle profiles of CRISPR/Cas9‐edited clones after 72 hours irradiation exposure (2 Gy). All the events placed right from the G2/M peak at 400 DNA content units were considered >4n population

Phenotypical analyses of these edited cell lines revealed that HG3‐del(11q) *ATM* ^MUT^ *TP53* ^MUT^ cells were markedly enlarged compared to the rest of the conditions (Figure 2E). Giemsa staining of these cell lines showed that these atypical cells had an irregular cytoplasm with the presence of degenerative vacuoles and were frequently multinucleated, suggesting a profound defect in mitosis. In fact, the mitotic index was found significantly lower in these cells than CLL cells without biallelic loss of *ATM* and *TP53* (Figure 2E; Table S6). 3‐(4,5‐dimethylthiazol‐2‐yl)‐2,5‐diphenyltetrazolium bromide (MTT) and growth assays corroborated impaired proliferation of these cells in comparison to HG3 *TP53* ^MUT^ cells (Figures 3A and 3B). Besides, cell‐cycle distribution analysis under basal conditions revealed the presence of increased G2/M and >4n events in HG3‐del(11q) *ATM* ^MUT^ *TP53* ^MUT^ clones (Figures 3D and 3E). Interestingly, these differences in proliferation were not related to an apoptotic defect, since PARP1 and caspase‐3 cleavage levels were similar between all cell lines (Figure 3C).

In order to determine how these CRISPR/Cas9‐generated cell lines responded to double strand breaks induction, cells were irradiated (IR), and cell cycle profiles were analyzed by measuring the DNA content after staining with propidium iodide (PI) 24, 48, and 72 hours after irradiation (Figures 3D and 3E). As expected, we found that HG3^WT^ and HG3‐del(11q) cells exhibited cell cycle arrest 24 hours post‐IR, but after that time cells had repaired their lesions and escaped the G2 arrest. In addition, HG3 *TP53* ^MUT^, and HG3‐del(11q) *TP53* ^MUT^ cells showed a G2/M cell cycle arrest in accordance with *TP53*‐defective cell‐cycle phenotype,^37^ which was also overcame 48 hours post‐IR. Conversely, HG3‐del(11q) *ATM* ^MUT^ *TP53* ^MUT^ cells exhibited a profound G2/M cell‐cycle arrest 24 hours post‐IR, with an increase in the number of events >4n, revealing the existence of polyploid cells in accordance with the presence of enlarged multinucleated cells even in the absence of exogenous DNA damage. The polyploid population (>4n), together with the persistence of G2/M arrest after irradiation (Figure 3E), further indicates the presence of mitotic defects in CLL cells harboring biallelic *ATM* and *TP53* defects.

### 3.4 *TP53* mutations can co‐exist with monoallelic del(11q), favoring in vivo clonal expansion of del(11q) CLL cells

Considering that *TP53* mutations or deletions were significantly enriched in the subset of monoallelic del(11q) CLL patients from our NGS analysis and significantly correlated with a worse prognosis, we next investigated the effects of the combination of these alterations in our CRISPR/Cas9‐engineered cell lines. We first interrogated whether the introduction of these alterations had an impact on CLL cell growth in vitro. Viability and growth assays revealed that the introduction of *TP53* mutations in HG3^WT^ cells resulted in increased proliferation (Figures 3A and 3B). In addition, HG3‐del(11q) *TP53* ^MUT^ cells had higher in vitro growth rates than HG3‐del(11q) cells. Notably, the introduction of an *ATM* truncating mutation in the remaining allele of these cell lines suppressed this proliferative advantage, since HG3‐del(11q) *ATM* ^MUT^ *TP53* ^MUT^ cells growth rates were comparable to those of HG3‐del(11q) cells (Figures 3A and 3B).

**FIGURE 4:**
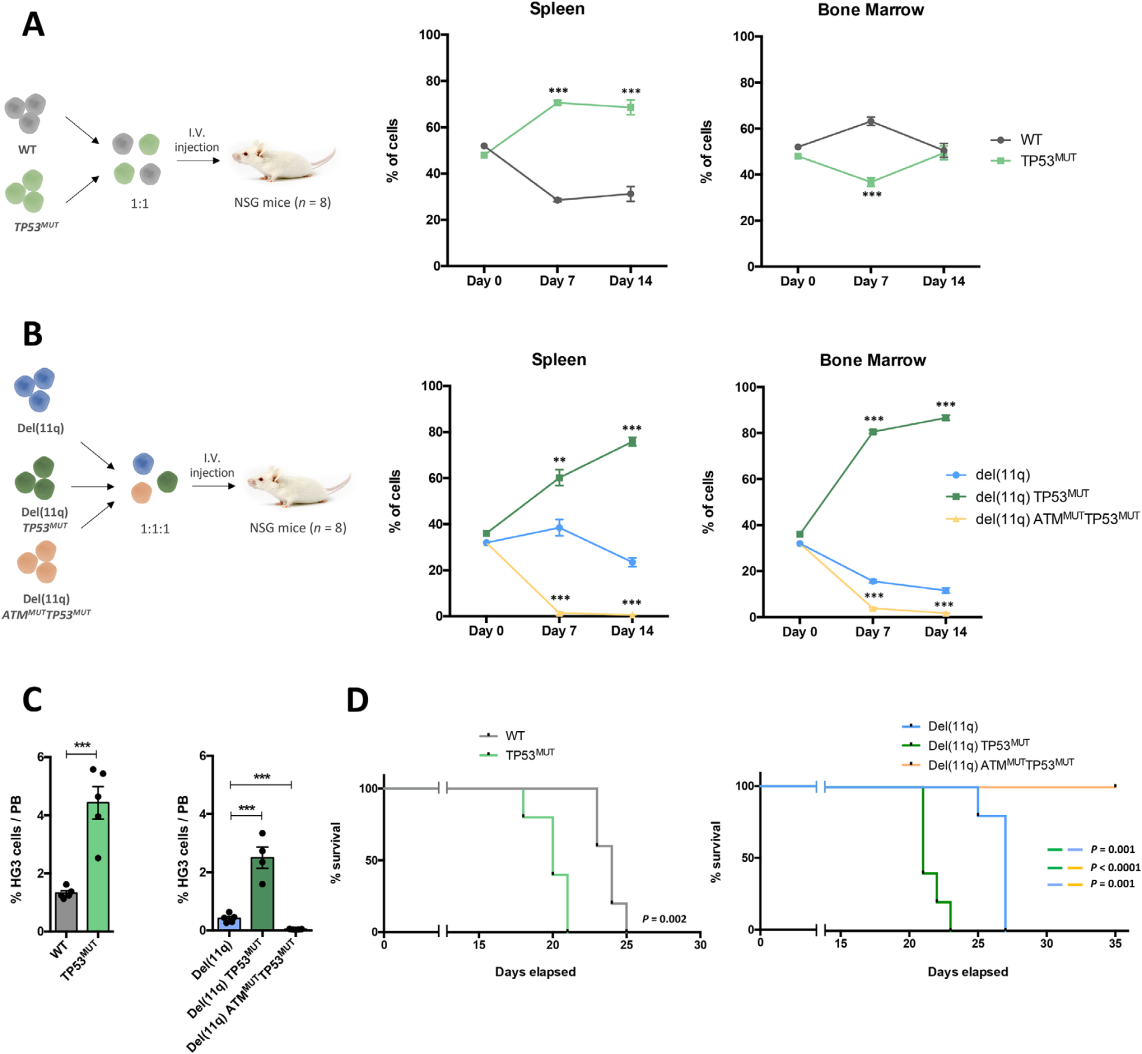
In vivo clonal competition analysis of xenotransplanted NSG mice. (A) HG3^WT^ GFP‐tagged and HG3 *TP53* ^MUT^ RFP‐tagged cells were mixed at a ratio 1:1 and injected into NSG mice (*n* = 8). Spleen and bone marrow infiltration were assessed by flow cytometry 7 (*n* = 4) and 14 (*n* = 4) days post‐injection. (B) HG3‐del(11q) RFP‐tagged, HG3‐del(11q) *TP53* ^MUT^ GFP‐tagged, and HG3‐del(11q) *ATM* ^MUT^ *TP53* ^MUT^ GFP, and RFP‐tagged cells were mixed at a ratio 1:1:1 and injected into NSG mice (*n* = 8). Spleen and bone marrow infiltration were assessed by flow cytometry 7 (*n* = 4) and 14 (*n* = 4) days post‐injection. Data are represented as the mean ± SD. p < 0.05 (*), p < 0.01 (**), p < 0.001 (***). (C) Quantification of GFP+ and/or RFP+ cell populations in the peripheral blood of HG3^WT^ and HG3 *TP53* ^MUT^ (left), and HG3‐del(11q), HG3‐del(11q) *TP53* ^MUT^, and HG3‐del(11q) *ATM* ^MUT^ *TP53* ^MUT^ (right) xenografted mice 17 days after intravenous injection. Data are shown as mean ± SEM. p < 0.001 (***). (D) Kaplan‐Meier overall survival curve of HG3^WT^ (*n* = 5) and HG3 *TP53* ^MUT^ (*n* = 5) xenografted mice (left panel) and HG3‐del(11q) (*n* = 5), HG3‐del(11q) *TP53* ^MUT^ (*n* = 5), and HG3‐del(11q) *ATM* ^MUT^ *TP53* ^MUT^ (*n* = 5) xenotransplants (right panel). The reported *p* value was calculated by using the Log‐rank test

In order to evaluate how monoallelic or biallelic *ATM* and/or *TP53* lesions contributed to the clonal dynamics of CLL in an in vivo setting, GFP and/or RFP‐tagged HG3^WT^ and HG3 *TP53* ^MUT^ cells, as well as HG3‐del(11q), HG3‐del(11q) *TP53* ^MUT^, and HG3‐del(11q) *ATM* ^MUT^ *TP53* ^MUT^ were mixed at a ratio 1:1 and injected into NSG mice (Figures 4A and 4B). Clonal evolution was assessed by quantifying the relative number of the different subclones in both the spleen and the bone marrow one and 2 weeks after cell injection. In the first subset of mice, clonal competition between HG3^WT^ and HG3 *TP53* ^MUT^ cells was assessed, showing that *TP53* ^MUT^ cells were able to rapidly outcompete WT cells in spleen (Figure 4A). Intriguingly, *TP53* ^MUT^ cells had a preferential engraftment toward spleen than bone marrow of xenotransplanted mice, since HG3^WT^ cell counts were higher in the bone marrow 1 week after injection. However, *TP53* ^MUT^ cells were able to progress at a higher growth rate in the bone marrow compartment in the following week (Figure 4A).

The second subset of mice was injected with HG3‐del(11q), HG3‐del(11q) *TP53* ^MUT^, and HG3‐del(11q) *ATM* ^MUT^ *TP53* ^MUT^ cells. At day 7, a marked clonal advantage of HG3‐del(11q) *TP53* ^MUT^ cells over HG3‐del(11q) in spleen was observed, being these differences even higher 14 days after injection (Figure 4B). Similar effects were observed in the bone marrow of these mice, being the growth dynamics of HG3‐del(11q) *TP53* ^MUT^ cells four‐fold faster than HG3‐del(11q) cells. Interestingly, HG3‐del(11q) *ATM* ^MUT^ *TP53* ^MUT^ cells failed to engraft and compete with HG3‐del(11q) and HG3‐del(11q) *TP53* ^MUT^ cells, almost disappearing from both spleen and bone marrow 2 weeks after cell injection (Figure 4B).

In an additional experiment, HG3‐edited clones were injected individually into NSG recipients (*n* = 5/group) to investigate the effects of clonal expansion of each cell line in mice survival. To evaluate the engraftment capacity of each condition in the peripheral blood, mice were bled at day 17 post‐injection, and blood cell counts were analyzed by flow cytometry. In concordance with the results observed in the clonal competition experiments, HG3 *TP53* ^MUT^ and HG3‐del(11q) *TP53* ^MUT^ xenotransplanted mice had higher percentage of leukemic cells than HG3^WT^ and HG3‐del(11q) xenotransplants, respectively, whereas the percentage of leukemic cells in mice xenografted with HG3‐del(11q) *ATM* ^MUT^ *TP53* ^MUT^ was almost negligible (Figure 4C). These observations correlated with an impact in OS, having HG3 *TP53* ^MUT^ and HG3‐del(11q) *TP53* ^MUT^ xenotransplants a significantly shorter OS than HG3^WT^ and HG3‐del(11q) mice, respectively (Figure 4D). Moreover, HG3‐del(11q) *ATM* ^MUT^ *TP53* ^MUT^ xenotransplants had the longest survival of all groups, being still alive at the end of the experiment (day 35, Figure 4D). Altogether, these results are consistent with the poorer prognosis observed in del(11q) patients harboring *TP53* alterations (Figure 1) and strongly reinforce the severe cell cycle defects in HG3‐del(11q) *ATM* ^MUT^ *TP53* ^MUT^ cells and therefore, the mutual exclusivity of biallelic *ATM* alterations and *TP53* loss in CLL patients.

### 3.5 Del(11q) *TP53* ^MUT^ CLL cells show incomplete responses to B cell receptor signaling inhibitors

**FIGURE 5:**
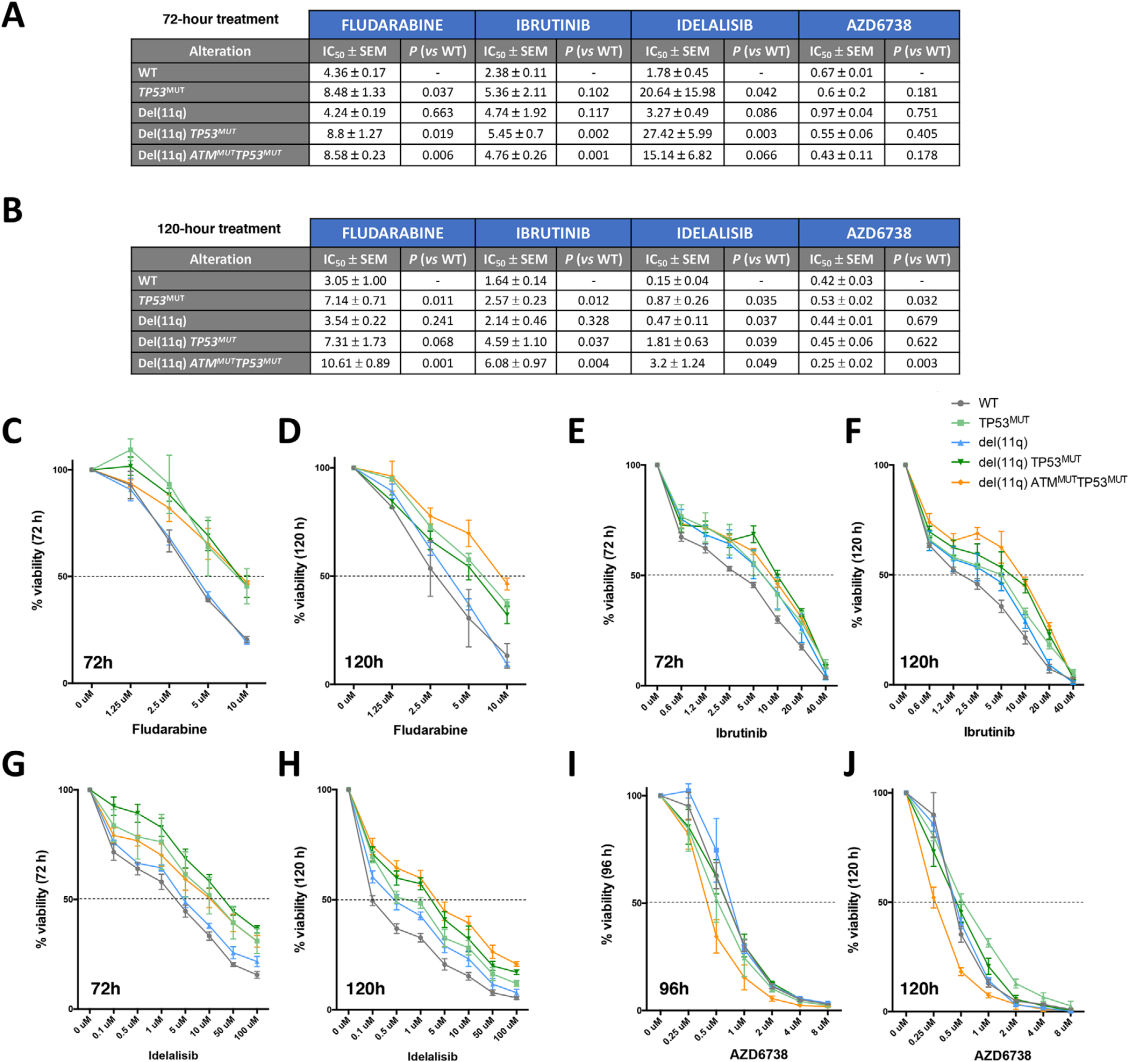
Cell viability studies of HG3‐edited clones in response to different drug treatments. (A and B) Tables indicating the IC_50_ ± SEM values of each CRISPR/Cas9‐edited HG3 cell line in response to fludarabine, ibrutinib, idelalisib or AZD6738, as well as the p‐values of the comparison between HG3^WT^ IC_50_ mean concentrations with the mean IC_50_ values from the rest of the conditions. (C‐J) HG3‐edited clones were treated with escalating doses of fludarabine (C and D), ibrutinib (E and F), idelalisib (G and H), and AZD6738 (I and J), and cell viability was assessed by MTT assay after the indicated treatment times. Surviving fraction is expressed relative to untreated controls. Data are summarized as the mean ± SD of three independent experiments

Given that *TP53* mutations seem to be a key determinant on disease progression for del(11q) CLL cells, we next evaluated drug responses of these isogenic CLL cell lines harboring high‐risk alterations. To validate that our CRISPR/Cas9‐generated cell lines could be used as models for treatment response, we tested the responses of all the cell lines to fludarabine since *TP53* mutations are a well‐known marker for fludarabine refractoriness.^22^, ^23^ We confirmed that truncated *TP53* was associated with fludarabine resistance independently of *ATM* status since HG3‐del(11q) *TP53* ^MUT^, as well as HG3 *TP53* ^MUT^ and HG3‐del(11q) *ATM* ^MUT^ *TP53* ^MUT^ cells showed consistently higher IC_50_ values compared to HG3^WT^ cells 72 hours after fludarabine treatment (mean IC_50_ 8.8, 8.48, 8.58 vs 4.36 μM, respectively) (Figures 5A and 5C). These results were also confirmed at longer fludarabine exposure times (Figures 5B and 5D).

Moreover, we tested responses of these cell lines to the novel targeted B cell receptor (BCR) signaling inhibitors such as ibrutinib and idelalisib, which were initially approved specifically for del(17p)/*TP53*‐mutated CLL patients. Notably, HG3‐del(11q) *TP53* ^MUT^ and HG3 *TP53* ^MUT^ cells showed a response to these drugs, although the IC_50_ values were still higher than the ones observed on the HG3^WT^ clones, especially in the case of idelalisib treatment (27.42 and 20.64 vs 1.78 μM, respectively, 72 hours after treatment) (Figures 5A, 5E, and 5G). HG3‐del(11q) *TP53* ^MUT^ and HG3 *TP53* ^MUT^ cells also showed higher IC_50_ values than HG3^WT^ cells after a 120‐hour exposure to these drugs (Figures 5B, 5F, and 5H).

Considering these results, we next prompted to assess whether this partial response of HG3‐del(11q) *TP53* ^MUT^ cells to ibrutinib and idelalisib could be overcome with the use of novel preclinical therapies. Since AZD6738, an Ataxia Telangiectasia and Rad3 related (ATR) serine/threonine protein kinase inhibitor, has been shown to induce synthetic lethality on *TP53*‐ or *ATM*‐defective CLL cells,^38^ we determined the response of our CRISPR/Cas9‐edited cell lines to this inhibitor. We found that HG3‐del(11q) *TP53* ^MUT^ cells were as sensitive to selective ATR inhibition as HG3^WT^ cells (Figures 5I and 5J), with a comparable 96‐hour treatment IC_50_ value between both conditions (mean IC_50_ 0.55 vs 0.67 μM).

## 4 DISCUSSION

Although the biological and prognostic impact of some individual CLL‐related alterations has been addressed in recent years,^4^, ^5^, ^16^, ^21^, ^22^, ^29^, ^30^, ^31^ most of these alterations usually co‐exist within the same tumoral clone, and how and which one of them cooperates with each other to drive leukemogenesis remains largely unknown. In this study, we explored the concurrence of monoallelic and biallelic del(11q)/*ATM* and *TP53* lesions by generating isogenic CRISPR/Cas9 in vitro models mimicking the genetic heterogeneity we observed in a high‐risk cohort of del(11q) CLL patients. Using this approach, we were able to determine the biological basis of the concurrence or mutual exclusivity of *TP53* alterations in del(11q) CLL.

Our targeted sequencing data of del(11q) CLL patients provide an understanding into the additional driver events accompanying this cytogenetic abnormality, highlighting that the vast majority of del(11q) patients harbor mutations in known CLL driver genes (Figure 1A), in contrast to what has been reported in del(13q) patients where 50% of them do not harbor any additional abnormalities.^4^, ^5^ Specifically, mutations in *ATM* and *BIRC3* in our study were significantly associated with the presence of del(11q), in accordance with previous studies showing that truncating mutations in these genes in del(11q) CLL patients result in a complete loss of functional ATM and BIRC3 proteins.^5^, ^10^, ^16^, ^39^ In addition, we also detected the presence of *TP53* lesions in a subset of del(11q) patients with a highly adverse clinical outcome. Indeed, *TP53* alterations were the only marker associated with a worse OS within the subgroup of del(11q) patients. Furthermore, we were able to recapitulate this combination of events (del(11q) *TP53* ^MUT^) in in vitro CLL models using the CRISPR/Cas9 editing system, showing that these cells have an in vivo clonal advantage over del(11q) cells without *TP53* alterations, offering a biological insight into the cooperation of these alterations in CLL progression and relapse. Taken together, our results suggest that the presence of *TP53* alterations in monoallelic del(11q) CLL patients may contribute to a negative predictive impact due to an increase competitive fitness of CLL clones harboring both of these alterations.

Large‐scale whole exome sequencing studies (WES) have revealed that genetic alterations in CLL do not randomly occur, and patterns of co‐occurrence or mutual exclusivity between these alterations have been suggested.^4^, ^5^, ^40^ However, the relationship between del(11q)/*ATM* and del(17p)/*TP53* lesions has not been well‐established. Previous reports have suggested that alterations in both of these genes tend to be mutually exclusive^18^, ^27^ whereas others have reported the concurrence of these alterations within the same tumoral clone.^20^, ^28^ In addition, WES studies from more than 1000 CLL samples were not able to identify any statistical evidence for a specific pattern of co‐occurrence or mutual exclusivity of *ATM* and *TP53* genetic alterations in CLL.^4^, ^5^ Our NGS and functional studies based on isogenic CRISPR/Cas9‐generated models indicate that co‐existence of these alterations within the same CLL clone robustly depends on the number of alleles affected by these events. We found that monoallelic del(11q) CLLs may harbor additional del(17p) and/or *TP53* mutations, whereas biallelic loss of *ATM* and *TP53* is virtually inexistent and therefore mutually exclusive in CLL patients. When we generated these scenarios in vitro, HG3 del(11q) *ATM* ^MUT^ *TP53* ^MUT^ cells showed abnormal phenotypic features such as mitotic impairment, leading to cell enlargement, polyploidy, and diminished proliferation capacity. Indeed, these cells failed to compete in vivo in xenotransplanted mice, being rapidly outcompeted by HG3‐del(11q) or HG3‐del(11q) *TP53* ^MUT^ cells. Thus, CRISPR/Cas9‐engineered CLL cell lines could be useful models not only to study the effects of individual or concurrent genetic abnormalities, but also to define the mechanisms underlying mutual exclusivity in order to find synthetic lethal interactions of clinical interest.

In the recent years, BCR kinase inhibitors such as ibrutinib and to a lesser extent idelalisib have revolutionized the treatment paradigm in CLL.^41^ Despite their proved benefits in comparison to previous chemotherapy‐based regimes in high‐risk cytogenetics patients, disease progression after treatment with these inhibitors has been increasingly reported.^42^, ^43^, ^44^ Our CRISPR/Cas9‐edited isogenic models have highlighted that del(11q) *TP53* ^MUT^ cell lines show only a partial response to ibrutinib or idelalisib, indicating that clones harboring these alterations might not be fully sensitive in a real‐life setting under therapy with these inhibitors. Although further studies in additional CLL cell lines with different genetic backgrounds would be required to validate these findings, our results are in line with observations made in ex vivo studies and clinical trials where del(17p)/*TP53* mutations are still a marker for less sensitivity and shorter OS in ibrutinib treated patients.^45^, ^46^ In the case of idelalisib, although the presence of del(17p)/*TP53* mutation did not show negative effects on clinical outcomes in a phase III trial of idelalisib in combination with rituximab,^47^ overall responses of *TP53*‐altered patients are still less frequent within this subgroup of patients.^48^, ^49^ Altogether, our results suggest that these CRISPR/Cas9‐edited CLL cell lines could be useful models to further predict treatment response of high risk del(11q) *TP53* ^MUT^ CLL cells, providing a pre‐clinical tool to explore novel therapeutic strategies such as ATR inhibitors in this subset of CLL cases.

In conclusion, this work addresses the biologic and prognostic implications of concurrent *TP53* alterations in del(11q) CLL. We show that mutations in *TP53* can appear in a subset of monoallelic del(11q) CLL cases, conferring clonal advantage in vivo, and therefore a dismal clinical impact in terms of OS in this subgroup of CLL patients. In addition, we also assess the biological basis of mutual exclusivity of biallelic *ATM* and *TP53* alterations in CLL, underscoring the importance of the number of alleles affected by these alterations in CLL, establishing novel pre‐clinical models for the study of the biology and therapeutic response of concurrent genetic abnormalities in the disease.

## CONFLICT OF INTEREST

The authors declare that there is no conflict of interest that could be perceived as prejudicing the impartiality of the research reported.

## ETHICS COMMITTEE APPROVAL

The present study was approved by the local ethics committee (Comité Ético de Investigación Clínica, Hospital Universitario de Salamanca). Written informed consent was obtained from all participants before they entered the study.

## AUTHOR CONTRIBUTIONS

Miguel Quijada‐Álamo designed experiments, performed CRISPR/Cas9 generation of engineered CLL cell lines, carried out functional studies, analyzed the data, and wrote the paper. Claudia Pérez‐Carretero designed experiments, performed sample selection, carried out NGS experiments, analyzed the data, and wrote the paper. María Hernández‐Sánchez designed CRISPR/Cas9 experiments and together with Ana‐Eugenia Rodríguez‐Vicente contributed to NGS experiments, data analysis, and interpretation of the results. Ana‐Belén Herrero designed DNA damage and repair experiments and contributed to data analysis. Jesús‐María Hernández‐Sánchez, Marta Martín‐Izquierdo, and Sandra Santos‐Mínguez performed NGS studies and data analysis. Mónica del Rey contributed in the revision experiments and analyses. Teresa González performed sample selection and provided clinical data. Araceli Rubio‐Martínez, Alfonso García de Coca, Julio Dávila‐Valls, and José‐Ángel Hernández‐Rivas provided patient samples and clinical data. Helen Parker and Jonathan C. Strefford provided biological and clinical data from the validation cohort. Rocío Benito contributed to data analysis and interpretation of the results. José‐Luis Ordóñez performed functional experiments and together with Jesús‐María Hernández‐Rivas conceived the study, designed the experiments, supervised the research, and critically reviewed the manuscript. All authors discussed the results and revised the manuscript.

## Supporting information

Supporting information.

## Data Availability

The data that support the findings of this study are available from the corresponding author upon reasonable request.
