# Supplementary material for: Dissecting the role of TP53 alterations in del(11q) chronic lymphocytic leukemia
Source: Clin Transl Med. 2021 Feb 4;11(2):e304. doi: 10.1002/ctm2.304 (PMC7862176; doi:10.1002/ctm2.304)
Supplement: Supplementary file 1 — Supporting information. [file CTM2-11-e304-s001.docx]

**SUPPLEMENTARY INFORMATION**

**Dissecting the role of *TP53* alterations in del(11q) chronic lymphocytic leukemia**

Miguel Quijada-Álamo, Claudia Pérez-Carretero, María Hernández-Sánchez, Ana-Eugenia Rodríguez-Vicente, Ana-Belén Herrero, Jesús-María Hernández-Sánchez, Marta Martín-Izquierdo, Sandra Santos-Mínguez, Mónica del Rey, Teresa González, Araceli Rubio-Martínez, Alfonso García de Coca, Julio Dávila-Valls, José-Ángel Hernández-Rivas, Helen Parker, Jonathan C. Strefford, Rocío Benito, José-Luis Ordóñez and Jesús-María Hernández-Rivas

This section contains:

1. Supplementary methods2
2. Supplementary Tables S1-66
3. Supplementary Figure S1-212

**SUPPLEMENTARY METHODS**

**Cell lines, culture conditions, drugs and reagents**

The human CLL-derived cell line HG3 was purchased from DMSZ (Deuthche Sammlung von Mikroorganismen and Zellkulturen) and was cultured in RPMI 1640 medium (Life Technologies) supplemented with 15% Fetal Bovine Serum (FBS), 1% glutaMAX and 1% penicillin/streptomycin (Life Technologies). HEK 293T cells for lentiviral production were obtained from DMSZ and maintained in DMEM (Life Technologies) supplemented with 10% FBS, 1% glutaMAX and 1% penicillin/streptomycin. All cell lines were incubated at 37ºC in a 5% CO_2_ atmosphere. The presence of mycoplasma was tested frequently with MycoAlert kit (Lonza), only using mycoplasma-free cells in all the experiments carried out.

Idelalisib and AZD6738 were obtained from Selleckchem, ibrutinib was from LC Laboratories and fludarabine was from Sigma. All drugs were resuspended in DMSO (Sigma).

**Fluorescence in situ hybridization (FISH)**

Interphase FISH was performed on peripheral blood or bone marrow samples using commercially available probes for the 13q14, CEP12, 11q22/*ATM*, 17p13/*P53* and 14q32 regions (Vysis, Abbott Laboratories, IL, USA). The methods used for FISH analysis have been described elsewhere.^1^ Signal screening was carried out in at least 200 cells with well-delineated fluorescent spots. In all cases, a score of ≥10% was considered positive, based on the cut-off value used by our laboratory.

**NGS data analysis and variant calling**

After the enrichment of interest regions*,* paired-end sequencing (151-bp reads) was run on the Illumina NextSeq instrument (Illumina). At the end of the process, this platform collects all the information in demultiplexed and paired FASTQ files to continue with bioinformatic analysis. SureSelect^QXT^ adaptor sequences were trimmed using SureCall (Agilent’s NGS data analysis software) and sequenced reads were aligned to the hg19 human reference genome (GRCh37/hg19). Coverage along the targeted regions was analyzed using BAM files generated by SAMtools^2^ from the FASTQ files.

We used GATK (Genome Analysis Toolkit),^3^ to generate the variant calling files (VCFs), and thereby to identify somatic substitutions or variant calling. The VCFs were annotated using Annovar^4^ and filtered by depth and variant allele frequency (VAF), position, and function of the variant in the coding sequence (hg19 refGene), in common polymorphism databases (dbSNP, 1000 genomes), in the cancer-specific database Catalogue of Somatic Mutations in Cancer (COSMIC) or in the Exome Aggregation Consortium (ExAC). Aligned reads were manually reviewed with the Integrative Genomics Viewer (IGV) to confirm and interpret variant calls and reduce the risk of false positives.^5^ For a deeper analysis we manually screened variants in VarSome, which integrates a wide range of population databases and pathogenicity predictors (gnomAD, ICGC, SIFT, Polyphen2, ClinVar, MutationAssesor, MutPred and FATHMM among others). Functional impact of *TP53* mutations described in this work was checked in IARC TP53 database. All mutations were annotated as non-functional with the exception of variant p.R290H, previously described as 'supertrans' or with high transcriptional activity.

**sgRNA cloning, lentiviral generation and transduction**

sgRNA cloning was carried out as previously described.^6^ The pLKO5 plasmids carrying the sgRNAs of interest were co-transfected in addition to pMD2.G (Addgene #12259) and psPAX2 (Addgene #12260) into HEK 293T using Lipofectamine 2000^®^ (Life Technologies). Supernatant containing the lentiviral particles was collected 48 and 72 hours after transfection and concentrated using Lenti-X concentrator^®^ (Clontech). HG3 cell transduction was carried out in the presence of polybrene (8 μg/mL) as previously described.^7^ pLKO5 vectors carrying the desired sgRNAs packed into lentiviral particles were then transduced into Cas9-expressing HG3 cells and single-cell flow-sorted clones were expanded and screened. Three to five different clones harboring loss-of-function mutations were chosen for each CRISPR-generated cell line to perform further functional studies.

**PCR and sequencing of sgRNA target sites**

Genomic DNA (gDNA) was extracted using the QIAampDNA Micro Kit (Qiagen) following the manufacturer’s instructions. PCR was performed using primers flanking the target sites for the sgRNAs (Supplemental Table S2) or the sgRNA off-target sites (Supplementary Table S3). For screening the loss-of-function mutations, PCR products were purified using High Pure PCR Product Purification Kit (Roche) and resulting indels at the expected locations were confirmed by Sanger sequencing. The efficiency of the sgRNAs was assessed by Tracking of Indels by Decomposition (TIDE) software (https://tide-calculator.nki.nl; Netherlands Cancer Institute).^8^

**Western blot analysis**

Cells were washed twice in PBS and lysed in ice-cold lysis buffer (50 nM TrisHCl pH 7.4, 150 mM NaCl, 1 mM EDTA, 1% Triton X-100) containing protease inhibitors (cOmplete^®^, Roche) and phosphatase inhibitors (PhosSTOP^TM^, Roche). Protein concentration was measured using the Bradford assay (BioRad). Protein samples were subjected to SDS-PAGE and transferred to a nitrocellulose membrane (GE Healthcare). After blockade, membranes were incubated with the following primary antibodies purchased from Cell Signaling Technologies: anti-ATM (#2873, Rabbit), anti-TP53 (#9282, Rabbit), anti-β-actin (#4967, Rabbit), anti-GAPDH (#5174, Rabbit), anti-PARP1 (#9542, Rabbit) and anti-caspase 3 (#9662, Rabbit). Horseradish peroxidase-linked anti-rabbit antibody (#7074, Cell Signaling Technologies) was used as secondary antibody at 1:5,000 dilution. Antibody signal was detected using ECL^TM^ Western Blotting Detection Reagents (RPN2209, GE Healthcare).

**Cell cycle analysis**

Cell distribution in the cell cycle phase was analyzed measuring the DNA content by propidium iodide (PI) labeling after cell permeabilization. In brief, cells were irradiated at a dose of 2 Gy during the exponential phase of cell growth with γ-rays using a Gammacell 1000 Elite irradiator (Cesium137). After irradiation, 3 x 10^5^ cells were seeded in 24-well plates and ethanol permeabilization and PI labeling were performed at different time points. DNA content was measured by flow cytometry.

**Viability and growth assays**

Cell viability was assessed using 3-(4,5-dimethylthiazol-2-yl)-2,5-diphenyltetrazolium bromide (MTT) colorimetric assay (Sigma-Aldrich). After drug treatment, cells were incubated for 2h with a 1:10 MTT solution and subsequently added 1:2 SDS-HCl in agitation for 6 hours. Absorbance was read on an Infinite® F500 Tecan plate reader (Tecan) at 570nm.

For the determination of the growth exponential curves of HG3 CRISPR/Cas9-edited clones, cells were seeded at a concentration of 3 x 10^4^ cells/mL and cell counts were assessed every 24 hours for a total of 5 days by Trypan Blue exclusion.

**REFERENCES**

1. González MB, Hernández JM, García JL, et al. The value of fluorescence in situ hybridization for the detection of 11q in multiple myeloma. *Haematologica*. 2004;89(10):1213-1218.

2. Li H, Handsaker B, Wysoker A, et al. The Sequence Alignment/Map format and SAMtools. *Bioinformatics*. 2009;25(16):2078-2079.

3. McKenna A, Hanna M, Banks E, et al. The genome analysis toolkit: A MapReduce framework for analyzing next-generation DNA sequencing data. *Genome Res*. 2010;20(9):1297-1303.

4. Wang K, Li M, Hakonarson H. ANNOVAR: Functional annotation of genetic variants from high-throughput sequencing data. *Nucleic Acids Res*. 2010;38(16).

5. Robinson JT, Thorvaldsdóttir H, Wenger AM, Zehir A, Mesirov JP. Variant Review with the Integrative Genomics Viewer. *Cancer Res*. 2017;77(21):e31-e34.

6. García-Tuñón I, Hernández-Sánchez M, Ordoñez JL, et al. The CRISPR/Cas9 system efficiently reverts the tumorigenic ability of BCR/ABL in vitro and in a xenograft model of chronic myeloid leukemia. *Oncotarget*. 2017;8(16):26027-26040.

7. Quijada-Álamo M, Hernández-Sánchez M, Alonso-Pérez V, et al. CRISPR/Cas9-generated models uncover therapeutic vulnerabilities of del(11q) CLL cells to dual BCR and PARP inhibition. *Leukemia*. 2020;34(6):1599-1612.

8. Brinkman EK, Chen T, Amendola M, van Steensel B. Easy quantitative assessment of genome editing by sequence trace decomposition. *Nucleic Acids Res*. 2014;42(22):e168.

| **Supplementary Table S1.** List of regions and mean coverage of genes included in the custom-designed panel of NGS. | | | | |
| --- | --- | --- | --- | --- |
| **Gene** | **Transcript** | **Regions** | **Mean coverage (reads/base)** |  |
| *ARID1A* | ENST00000324856 | Exons 1-20 | 814 |  |
| *ASXL1* | ENST00000375687 | Exons 1-13 | 701 |  |
| *ATM* | ENST00000278616 | Exons 2-63 | 456 |  |
| *ATRX* | ENST00000373344 | Exons 1-35 | 243 |  |
| *BAX* | ENST00000345358 | Exon 2-6 | 826 |  |
| *BAZ2A* | ENST00000551812 | Exons 2-28 | 819 |  |
| *BCL2* | ENST00000333681 | Exons 2-3 and 5'UTR | 536 |  |
| *BCOR* | ENST00000378444 | Exons 2-15 | 398 |  |
| *BIRC3* | ENST00000263464 | Exons 2-9 | 483 |  |
| *BRAF* | ENST00000288602 | Exons 11-16 | 499 |  |
| *BTK* | ENST00000308731 | Exons 2-19 | 456 |  |
| *CARD11* | ENST00000396946 | Exons 3-17 | 795 |  |
| *CCND2* | ENST00000261254 | Exons 1-5 | 827 |  |
| *CD19* | ENST00000324662 | Exons 1-6 | 845 |  |
| *CDC73* | ENST00000367435 | Exons 1-16 | 436 |  |
| *CHD2* | ENST00000394196 | Exons 2-39 | 463 |  |
| *DDX3X* | ENST00000399959 | Exons 1-16 | 376 |  |
| *EGR2* | ENST00000242480 | Exons 1-2 | 1021 |  |
| *FAM50A* | ENST00000393600 | Exons 2-12 | 478 |  |
| *FAT1* | ENST00000441802 | Exons 2-27 | 754 |  |
| *FBXW7* | ENST00000281708 | Exons 7-12 | 721 |  |
| *FUBP1* | ENST00000370768 | Exons 1-19 | 451 |  |
| *HIST1H1B* | ENST00000331442 | Exon 1 | 954 |  |
| *HIST1H1E* | ENST00000304218 | Exon 1 | 668 |  |
| *IGLL5* | ENST00000526893 | Exons 1-3, 5'UTR | 555 |  |
| *IKZF3* | ENST00000346872 | Exon 5 | 696 |  |
| *IRF4* | ENST00000380956 | Exons 2-9 | 698 |  |
| *KLHL6* | ENST00000341319 | Exons 1-7 | 693 |  |
| *KRAS* | ENST00000311936 | Exons 2-5 | 543 |  |
| *MAP2K1* | ENST00000307102 | Exons 1-11 | 561 |  |
| *MED12* | ENST00000374080 | Exons 1-4 | 389 |  |
| *MGA* | ENST00000570161 | Exons 2-23 | 572 |  |
| *MYD88* | ENST00000396334 | Exons 2-5 | 781 |  |
| *NFKBIE* | ENST00000275015 | Exons 1-2 | 969 |  |
| *NOTCH1* | ENST00000277541 | Exon 34 and 3'UTR | 1208 |  |
| *NRAS* | ENST00000369535 | Exons 2-3 | 587 |  |
| *NXF1* | ENST00000294172 | Exons 3-21 | 653 |  |
| *PAX5* | ENST00000358127 | enhancer | 791 |  |
| *PCDH10* | ENST00000264360 | Exons 1-5 | 562 |  |
| *PIK3CD* | ENST00000377346 | Exons 3-24 | 848 |  |
| *PLCG2* | ENST00000564138 | Exons 2-33 | 618 |  |
| *POT1* | ENST00000357628 | Exons 5-19 | 405 |  |
| *PTPN11* | ENST00000351677 | Exonso 2-15 | 645 |  |
| *RPS15* | ENST00000593052 | Exons 2-4 | 679 |  |
| *SAMHD1* | ENST00000262878 | Exons 1-16 | 465 |  |
| *SETD2* | ENST00000409792 | Exons 1-21 | 476 |  |
| *SF3B1* | ENST00000335508 | Exons 14-16 | 524 |  |
| *SORCS2* | ENST00000507866 | Exons 1-27 | 876 |  |
| *TP53* | ENST00000269305 | Exons 4-10 | 634 |  |
| *TRAF3* | ENST00000392745 | Exons 1-12 | 641 |  |
| *XPO1* | ENST00000401558 | Exons 15-16 | 556 |  |
| *ZC3H18* | ENST00000301011 | Exons 2-18 | 780 |  |
| *ZMYM3* | ENST00000373998 | Exons 2-25 | 507 |  |
| *ZNF292* | ENST00000339907 | Exons 1-8 | 401 |  |

**Supplementary Table S2.** Oligos designed for each sgRNA and PCR primers of sgRNA target sites.

| **Target** | **Forward (5’-3’)** | **Reverse (5’-3’)** |
| --- | --- | --- |
| *ATM* exon 10 sgRNA1 | CACCG**GTAAGGCATCGTAACACATA** | AAAC**TATGTGTTACGATGCCTTAC**C |
| *ATM* exon 10 sgRNA2 | CACCG**GACACAATGCAACTTCCGTA** | AAAC**TACGGAAGTTGCATTGTGTC**C |
| *TP53* exon 4 sgRNA1 | CACCG**CCATTGTTCAATATCGTCCG** | AAAC**CGGACGATATTGAACAATGG**C |
| *TP53* exon 4 sgRNA2 | CACCG**CCCCGGACGATATTGAACAA** | AAAC**TTGTTCAATATCGTCCGGGG**C |
| Control sgRNA1 | CACCG**ACGGAGGCTAAGCGTCGCAA** | AAAC**TTGCGACGCTTAGCCTCCGT**C |
| *ATM* exon 10 (PCR) | TCCTGCCAATTTAGGAAGTAGGAC | CTGCAGGCTGACCCAGTAAA |
| *TP53* exon 4 (PCR) | AGACCTGTGGGAAGCGAAAA | GACAGGAAGCCAAAGGGTGA |

**Supplementary Table S3**. Predicted off-target regions and oligos designed for PCR and Sanger sequencing.

| **sgRNA sequence** | **Off-target sequence** | **Gene name** | **Ensembl ID** | **# mismatches** | **Chromosome** | **Exon/Intron** | **Forward Primer (5'-3')** | **Reverse Primer (5'-3')** |
| --- | --- | --- | --- | --- | --- | --- | --- | --- |
| ATM ex10 sgRNA1  **GTAAGGCATCGTAACACATA** | **t**TAAGG**t**AT**g**GTAA**a**ACATA-AGG | NRXN1 | [ENSG00000179915](http://feb2014.archive.ensembl.org/Homo_sapiens/Gene/Summary?db=core;g=ENSG00000179915) | 4 | 2 | Intron 15-16 | TGCAAGATCTATGTTGAGGCCA | CTGAGAATCGAGCGGTGTCA |
|  | **a**TAAGGC**ta**CGTA**t**CACATA-GGG | BICDL1 | [ENSG00000135127](http://feb2014.archive.ensembl.org/Homo_sapiens/Gene/Summary?db=core;g=ENSG00000135127) | 4 | 12 | Intron 2-3 | TAGCTCCACTTGCTGTGTGTT | TTGACGCAAGGGCATTTCTC |
|  | **t**TAAGGC**c**T**tt**TAACACATA-GGG | NRAP | [ENSG00000197893](http://feb2014.archive.ensembl.org/Homo_sapiens/Gene/Summary?db=core;g=ENSG00000197893) | 4 | 10 | Intron 22-23 | CCAGGTGGACTAGAGAGGCT | AAGACGCAGCTCAAACCCTT |
| ATM ex10 sgRNA2  **GACACAATGCAACTTCCGTA** | GA**gta**AA**a**GCAACTTCCGTA-GGG | NBPF3 | [ENSG00000142794](http://feb2014.archive.ensembl.org/Homo_sapiens/Gene/Summary?db=core;g=ENSG00000142794) | 4 | 1 | Exon 13 | CAGAGAGCTGCCGGAGGTAG | GAATCAGAGTGCCACAGGCAT |
|  | **c**A**g**A**g**AA**g**GCA**g**CTTCCGTA-AGG | ZNF407 | [ENSG00000215421](http://feb2014.archive.ensembl.org/Homo_sapiens/Gene/Summary?db=core;g=ENSG00000215421) | 5 | 18 | Intron 7-8 | AATCAGCCCGCTGAAAACGG | AACGAAATCAGAGCCCTGCC |
|  | **a**A**g**ACAA**g**GCA**c**CT**g**CCGTA-TGG | ZNF704 | [ENSG00000164684](http://feb2014.archive.ensembl.org/Homo_sapiens/Gene/Summary?db=core;g=ENSG00000164684) | 5 | 8 | Intron 2-3 | TGTAGTGCATGCCTGAGGGG | TTGTGCAGGTGGTAACTGCG |
| TP53 ex4 sgRNA1  **CCATTGTTCAATATCGTCCG** | **aa**AT**ca**TTC**c**ATATCGTCCG-CGG | PTPN3 | [ENSG00000070159](http://feb2014.archive.ensembl.org/Homo_sapiens/Gene/Summary?db=core;g=ENSG00000070159) | 5 | 9 | 5' upstream seq | CTCCTGAAATGAGAGCCACGA | CTCGGCTTTCAAAGATGGCA |
|  | **g**CA**a**TGTTCA**gc**A**g**CGTCCG-TGG | TMEM132C | [ENSG00000181234](http://feb2014.archive.ensembl.org/Homo_sapiens/Gene/Summary?db=core;g=ENSG00000181234) | 5 | 12 | Intron 1-2 | AGACAGCAGGGGATGAAAACC | TTCAAGTGCCTCATTCCCACA |
|  | CCA**gg**G**aa**CAATA**g**CGTCCG-CGG | THSD7A | [ENSG00000005108](http://feb2014.archive.ensembl.org/Homo_sapiens/Gene/Summary?db=core;g=ENSG00000005108) | 5 | 7 | 5' UTR | TGAGGACAGTTGCCTCCG | TCTTCCACAGATAGAGGGTGGG |
| TP53 ex4 sgRNA2  **CCCCGGACGATATTGAACAA** | **a**CCC**a**GA**g**GATATT**t**AACAA-GGG | SUN3 | [ENSG00000164744](http://feb2014.archive.ensembl.org/Homo_sapiens/Gene/Summary?db=core;g=ENSG00000164744) | 4 | 7 | Intron 10-11 | CAGTTCAGTATGGCGACGCA | TGTTTGAACGGTGGTTCCTGT |
|  | CCC**t**GGA**gct**TATTGAACAA-TGG | NAV2 | [ENSG00000166833](http://feb2014.archive.ensembl.org/Homo_sapiens/Gene/Summary?db=core;g=ENSG00000166833) | 4 | 11 | 5' upstream seq | CACTGGACATCCCTATGCCG | CCTTGGCACATCCAGAGAGC |
|  | CCC**a**GGAC**cta**ATTGAACAA-GGG | EXOC3 | [ENSG00000180104](http://feb2014.archive.ensembl.org/Homo_sapiens/Gene/Summary?db=core;g=ENSG00000180104) | 4 | 5 | Exon 4 | GCATGCAGGCAGCCTTTTAT | GCCAGTTTGCTTTTTCCGGT |

**Supplementary Table S4.** Overall survival (OS) of del(11q) CLL cases according to the presence of additional genetic alterations (*n* = 47).

|  | **Yes (median OS, months)** | **No (median OS, months)** | ***P*^a^** |
| --- | --- | --- | --- |
| **Nº of mutated genes >2** | 67 | 66 | 0.729 |
| ***ATM*^MUT^** | 67 | 33 | 0.286 |
| ***NOTCH1*^MUT^** | 27 | 67 | 0.547 |
| ***SF3B1*^MUT^** | 66 | 65 | 0.352 |
| ***BIRC3*^MUT^** | 33 | 67 | 0.268 |
| ***DDX3X*^MUT^** | 24 | 66 | 0.301 |
| ***TP53*^MUT^** | 15 | 88 | **0.0001** |
| **Del(17p)** | 19 | 88 | **0.01** |
| ***TP53* alteration (deletion/mut)** | 17 | 88 | **0.0004** |
| **Monoallelic *TP53* loss** | 17 | 88 | **0.01** |
| **Biallelic *TP53* loss** | 11 | 88 | **0.001** |

^a^ Log-rank Test

**Supplementary Table S5.** List of CRISPR/Cas9-generated indels in *ATM*, *TP53* and off-target regions.

| **HG3 Clone** | **sgRNA** | **Indels in *TP53*** | **Indels in *ATM*** | **Off-target indels** |
| --- | --- | --- | --- | --- |
| WT Clone #1 | Control sgRNA1 | WT/WT | WT/WT | None |
| WT Clone #2 | Control sgRNA1 | WT/WT | WT/WT | None |
| WT Clone #3 | Control sgRNA1 | WT/WT | WT/WT | None |
| *TP53*^MUT^ Clone #1 | *TP53* ex4 sgRNA1 | +1/+1 | WT/WT | None |
| *TP53*^MUT^ Clone #2 | *TP53* ex4 sgRNA2 | +1/+1 | WT/WT | None |
| *TP53*^MUT^ Clone #3 | *TP53* ex4 sgRNA2 | +1/+1 | WT/WT | None |
| Del(11q) Clone #1 | Control sgRNA1 | WT/WT | Del(11q)/WT | None |
| Del(11q) Clone #2 | Control sgRNA1 | WT/WT | Del(11q)/WT | None |
| Del(11q) Clone #3 | Control sgRNA1 | WT/WT | Del(11q)/WT | None |
| Del(11q) *TP53*^MUT^ Clone #1 | *TP53* ex4 sgRNA2 | -7/+1 | Del(11q)/WT | None |
| Del(11q) *TP53*^MUT^ Clone #2 | *TP53* ex4 sgRNA2 | -8/+1 | Del(11q)/WT | None |
| Del(11q) *TP53*^MUT^ Clone #3 | *TP53* ex4 sgRNA2 | +1/+1 | Del(11q)/WT | None |
| Del(11q) *TP53*^MUT^ Clone #4 | *TP53* ex4 sgRNA2 | -7/-7 | Del(11q)/WT | None |
| Del(11q) *TP53*^MUT^ Clone #5 | *TP53* ex4 sgRNA2 | -4/-4 | Del(11q)/WT | None |
| Del(11q) *ATM*^MUT^*TP53*^MUT^ Clone #1 | *TP53* ex4 sgRNA2;  *ATM* ex10 sgRNA2 | +1/+1 | Del(11q)/+1 | None |
| Del(11q) *ATM*^MUT^*TP53*^MUT^ Clone #2 | *TP53* ex4 sgRNA2;  *ATM* ex10 sgRNA2 | -302/-302 | Del(11q)/+1 | None |
| Del(11q) *ATM*^MUT^*TP53*^MUT^ Clone #3 | *TP53* ex4 sgRNA2;  *ATM* ex10 sgRNA2 | +1/-7 | Del(11q)/-14 | None |
| Del(11q) *ATM*^MUT^*TP53*^MUT^ Clone #4 | *TP53* ex4 sgRNA2;  *ATM* ex10 sgRNA2 | -7/-7 | Del(11q)/+1 | None |
| Del(11q) *ATM*^MUT^*TP53*^MUT^ Clone #5 | *TP53* ex4 sgRNA2;  *ATM* ex10 sgRNA2 | +1/-11 | Del(11q)/-22 | None |

**Supplementary Table S6.** Phenotypical characteristics of Giemsa-stained CRISPR/Cas9-generated CLL cell lines.

|  | **Presence of pleomorphic cells (%)** | **Mitotic index**  **(per 40X field)** | **Presence of degenerative vacuoles** |
| --- | --- | --- | --- |
| **HG3^WT^** | No | 4-5 | No |
| **HG3 *TP53*^MUT^** | < 1 % | 4-5 | No |
| **HG3 del(11q)** | No | 3-4 | No |
| **HG3 del(11q) *TP53*^MUT^** | < 1% | 5-6 | No |
| **HG3 del(11q) *ATM*^MUT^*TP53*^MUT^** | > 5% | 1-2 | Yes |

**Supplementary Figure S1**

**
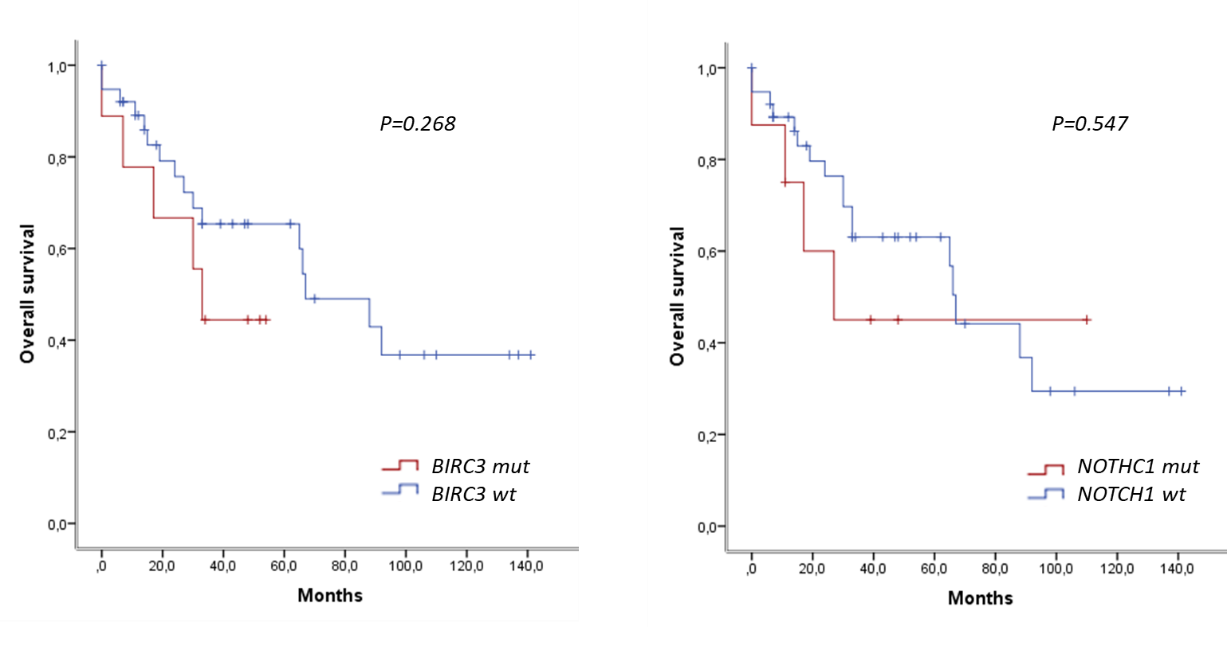
**

**
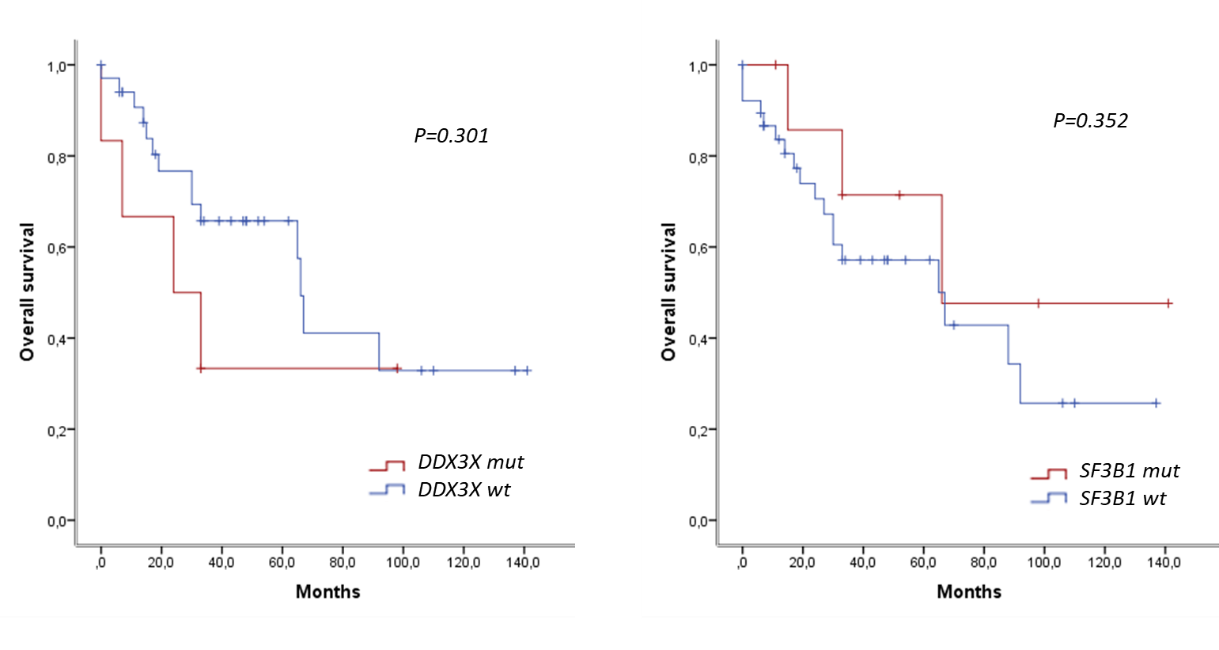
**

**
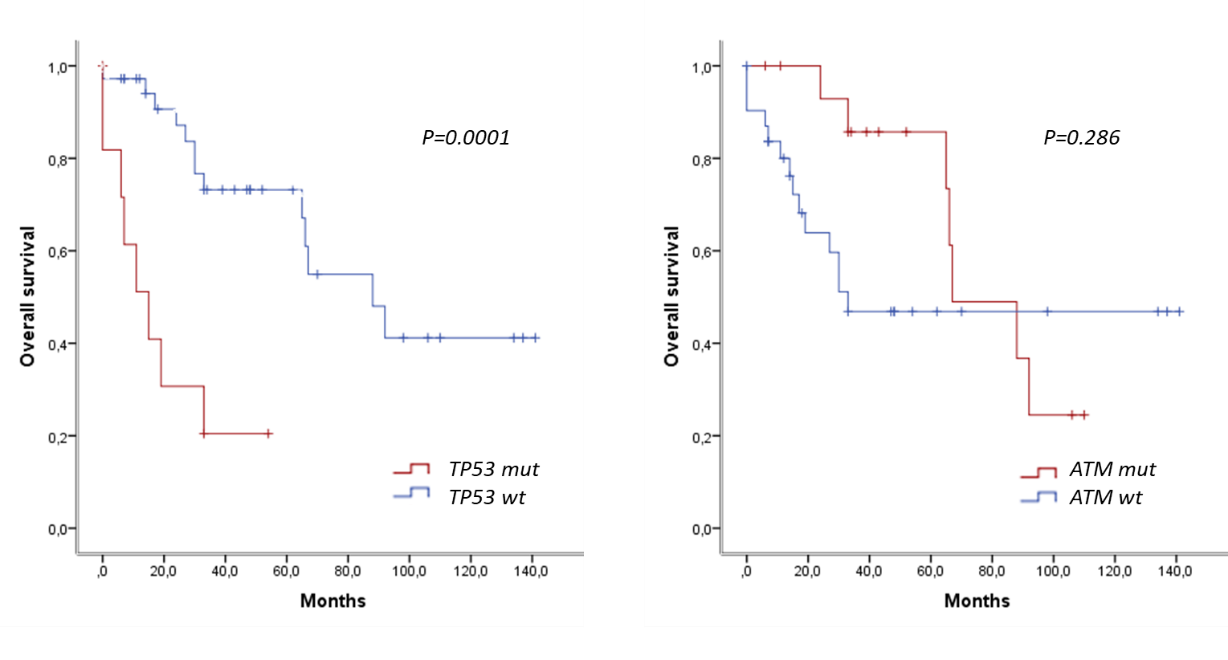
**

**Impact of *ATM*, *BIRC3*, *NOTCH1,* *DDX3X* and *SF3B1* mutations in the survival of del(11q) CLL patients.** Median overall survival (months) and *p*-values are shown in Supplementary Table S4**.**

**Supplementary Figure S2**

**A**

**
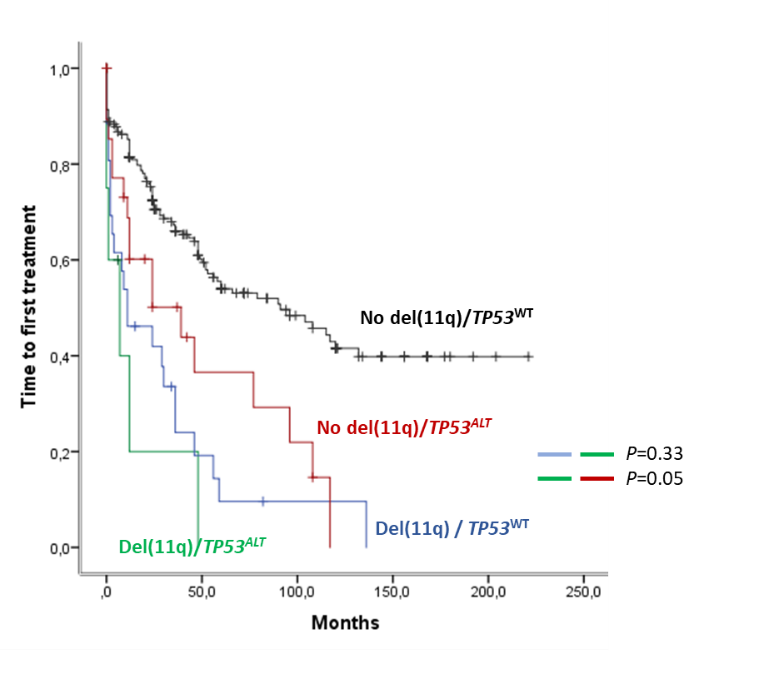

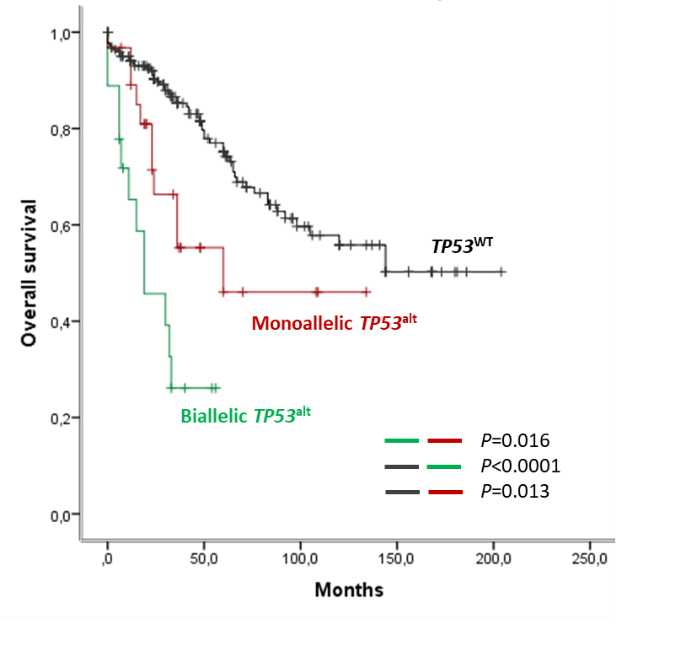
**

**B**

**Impact of *TP53* alterations on (A) overall survival of CLL patients and on (B) time to first treatment according to the presence of additional del(11q) (*n* = 271).**
